# Systemic Monocytic-MDSCs Are Generated from Monocytes and Correlate with Disease Progression in Breast Cancer Patients

**DOI:** 10.1371/journal.pone.0127028

**Published:** 2015-05-20

**Authors:** Caroline Bergenfelz, Anna-Maria Larsson, Kristoffer von Stedingk, Sofia Gruvberger-Saal, Kristina Aaltonen, Sara Jansson, Helena Jernström, Helena Janols, Marlene Wullt, Anders Bredberg, Lisa Rydén, Karin Leandersson

**Affiliations:** 1 Center for Molecular Pathology, Jan Waldenströms gata 59, Skåne University Hospital (SUS), Lund University (LU), 20502 Malmö, Sweden; 2 Translational Cancer Research, LU, Medicon Village 22381 Lund, Sweden; 3 Division of Oncology and Pathology, Department of Clinical Sciences Lund, LU, 22185 Lund, Sweden; 4 Department of Infectious Diseases, SUS, LU, 20502 Malmö, Sweden; 5 Medical Microbiology, Jan Waldenströms gata 59, SUS, LU, 20502 Malmö, Sweden; 6 Division of Surgery, Department of Clinical Sciences LU, 22185 Lund, Sweden; Sudbury Regional Hospital, CANADA

## Abstract

Myeloid-derived suppressor cells (MDSCs) are highly immunosuppressive myeloid cells, which increase in cancer patients. The molecular mechanism behind their generation and function is unclear. Whereas granulocytic-MDSCs correlate with poor overall survival in breast cancer, the presence and relevance of monocytic-MDSCs (Mo-MDSCs) is unknown. Here we report for the first time an enrichment of functional blood Mo-MDSCs in breast cancer patients before they acquire a typical Mo-MDSC surface phenotype. A clear population of Mo-MDSCs with the typical cell surface phenotype (CD14^+^HLA-DR^low/-^CD86^low/-^CD80^low/-^CD163^low/-^) increased significantly first during disease progression and correlated to metastasis to lymph nodes and visceral organs. Furthermore, monocytes, comprising the Mo-MDSC population, from patients with metastatic breast cancer resemble the reprogrammed immunosuppressive monocytes in patients with severe infections, both by their surface and functional phenotype but also at their molecular gene expression profile. Our data suggest that monitoring the Mo-MDSC levels in breast cancer patients may represent a novel and simple biomarker for assessing disease progression.

## Introduction

Immune cells constantly monitor the body to eliminate nascent transformed cells, a process known as immunosurveillance [1, 2]. As a tumor progresses however, the immune response is modulated by the tumor, resulting in non-responsiveness towards the tumor cells. The presence of local immunosuppressive cells correlate with poor prognosis in various forms of malignancies [3–9]. These populations contribute to a local immunosuppression at the site of the tumor [10]. The function of systemic immune cells in the peripheral blood of breast cancer patients, however, remains relatively unexplored.

Recently, much focus has been put on the myeloid-derived suppressor cells (MDSCs) that are frequently enriched in cancer patients [11]. Although poorly characterized in humans, MDSCs are typically described as immature myeloid cells with immunosuppressive properties and of either granulocytic- (G-MDSC; CD33^+^Lin^-^) or monocytic- (Mo-MDSC; CD14^+^HLA-DR^low/-^Co-receptor^low/-^) lineages [11]. The presence of granulocytic-MDSCs has been correlated with disease progression in many forms of cancer, including breast cancer [11–13]. Recent studies have identified an enrichment of Mo-MDSCs in the peripheral blood of melanoma, prostate cancer, glioblastoma, and bladder cancer patients [14–17]. It was even suggested that the immunosuppressive properties of MDSCs are attributed specifically to the peripheral blood MDSCs rather than the local, tumor-associated, MDSCs, emphasizing the importance of circulating MDSCs [18]. Whether this Mo-MDSC population is present in the peripheral blood of breast cancer patients remains to be determined. Furthermore, while local induction of MDSCs has been extensively investigated and involves tumor-/stroma-derived factors such as GM-CSF, IL-10, TGFβ, VEGF and PGE_2_, the origin and mechanism of generation of circulating Mo-MDSCs is, as of yet, largely unknown [11, 19–22].

Although originally described in cancer patients, MDSCs have also been shown to expand in the peripheral blood during other inflammatory conditions such as sepsis (*i*.*e*. an acute systemic inflammatory condition triggered by an infection) [11, 23]. During recent years it has become apparent that neoplastic and infectious diseases induce similar immune responses (e.g. reduced T cell activity and induction of regulatory T cells and MDSCs) [24]. In sepsis a rapid activation of innate immune cells occurs in order to eliminate the source of danger (systemic inflammatory response; SIRS). In parallel, an antagonistic anti-inflammatory and tissue regenerating response is activated in order to dampen the inflammatory reaction and induce healing once the threat is removed (compensatory anti-inflammatory response; CARS). Importantly, CARS-monocytes have a Mo-MDSC phenotype (CD14^+^HLA-DR^low/-^Co-receptor^low/-^) [25–27]. There are two hypotheses as to how CARS-monocytes are generated in sepsis patients: i) export of immature cells into the blood stream caused by an emergency myelopoiesis ii) a reprogramming of already exported monocytes [23, 25]. We have previously proposed that emergency myelopoiesis is the primary cause of granulocytic-MDSCs generation in sepsis patients [28]. Although emergency myelopoiesis also could be a likely explanation for Mo-MDSCs in sepsis, the classic hypothesis has been that monocytes in CARS patients promptly become reprogrammed towards an immunosuppressive state known as “endotoxin tolerance” [25, 26]. These reprogrammed monocytes also have the same phenotype and function as Mo-MDSCs (CD14^+^HLA-DR^low/-^Co-receptor^low/-^) [26, 27].

Today, knowledge is lacking as to whether a localized primary tumor can affect the systemic immune system or if this occurs only in disseminated disease. To investigate this we analyzed the leukocyte populations present in the peripheral blood of breast cancer patients at various stages of disease, but before start of adjuvant or palliative systemic treatments. Specifically, we examined the presence and function of circulating monocytes and Mo-MDSCs with the aim to investigate the clinical relevance and origin of Mo-MDSCs in breast cancer patients. We report that Mo-MDSCs significantly increase in the peripheral blood of breast cancer patients with locoregional recurrence or metastatic breast cancer (LRR/MBC) and correlate with increased metastasis to lymph nodes and visceral organs, suggesting that circulating Mo-MDSCs are a potential biomarker for disease progression. Surprisingly, monocytes enriched from patients with primary breast cancer without metastasis tend to exhibit immunosuppressive properties without observed changes in surface phenotype. Using gene expression profiling we could further show that monocytes/Mo-MDSCs from breast cancer patients with metastatic disease display a significant similarity to monocytes isolated from patients with sepsis, a disease displaying immunosuppressive monocytes. These similarities were not observed with monocytes from healthy controls or tuberculosis patients. We propose that systemic Mo-MDSCs are induced early during tumor progression, prior to induction of surface phenotype alterations. The Mo-MDSC surface phenotype changes appear as the disease progresses. This suggests that leukocytes are affected by the growing tumor prior to extravasation into the tumor tissue thus opening for the possibility of immune intervention as a therapeutic strategy in early breast cancer. Finally, our phenotypic and molecular findings suggest that the generation of Mo-MDSCs is similar in breast cancer and sepsis patients.

## Materials and Methods

### Patient samples

Peripheral blood was collected from 25 breast cancer patients diagnosed with stage IV breast cancer (locoregional recurrence or metastatic disease, LRR/MBC; mean age ±SD, 60±10 years; 100% female), 10 disparate patients with primary breast cancer without distant metastasis (mean age ±SD; 61±8 years, 100% female) and 13 healthy controls (HC, mean age ±SD; 38±14 years, 15% male and 85% female). All blood samples (1.5–2.0 mL for breast cancer patients and 4–5 mL for healthy donors) were collected at time of diagnosis before administration of adjuvant or palliative systemic therapy. For detailed patient information, see S1 Table. Peripheral blood (4–5 mL) from patients with gram-negative sepsis (18 patients, mean age +/-SD; 69+/-21 years, 33% male and 67% female) was used as a control for acute systemic immune response and immunosuppressive monocytes. The sepsis diagnosis was based on a combination of clinical symptoms and conventional testing using Swedish national QC approved culture. In addition, 6 patients with active tuberculosis were added as a control for a local chronic infection (mean age ±SD, 53±16 years; 67% male 33% female).

All blood samples were collected in EDTA-coated tubes and analyzed within 24h. Briefly, the peripheral blood was diluted 1:2 in phosphate-buffered saline (PBS; EDTA/sucrose), overlaid on Ficoll-Paque Plus (GE Healthcare, Uppsala, Sweden) and centrifuged at 400g, at room temperature, for 30 min with brake off. The peripheral blood mononuclear cells (PBMCs) were collected in PBS (EDTA/sucrose) and centrifuged at 350g, at 4°C, for 7 min (with brake on). Permission has been obtained from the Research Ethics Committee at Lund University Dnr 2012/689 for healthy blood donors, Dnr LU 75–02, LU 37–08, LU-658-09, LU 58–12 and LU 379–12 for patients with early breast cancer, Dnr 2010/1352 and Dnr 2011/748 for patients with LRR/MBC, and Dnr 288/2007 for sepsis and tuberculosis patients, respectively. All participating patients gave written informed consent.

### Flow cytometry

PBMCs (10 000–50 000 cells) and isolated monocytes (5000–10 000 cells) were immediately stained for flow cytometry for a total of 20 min at 4°C. Due to inadequate sample amount, we were not able to perform all flow cytometric analyses on all patients. Antibodies used; CD14 clone M5E2 (1:10), HLA-DR clone G46-6 (1:50), CD80 clone L307.4 (1:15), CD86 clone IT2.2 (1:15), CD83 clone HB15e (1:15), CD33 clone WM53 (1:10), CD163 clone GHI/61 (1:15), CD16 clone 3G8 (1:20), CD3 clone HIT3a (1:25), CD4 clone RPA-T4 (1:25), CD8 HIT8a (1:25), CD25 clone 2A3 (1:10), CD127-biotin clone HIL-7R-M21 (1:10), CD56 clone B159 (1:10), all from BD Biosciences. Cells were analyzed using a FACSCalibur (BD Biosciences, San Jose, CA, USA). Analyzes were performed gated on PBMCs (≥2000 events per sample) and using 7AAD dead exclusion stain (BD Biosciences). Blood dendritic cell analyzes were performed using Blood DC enumeration kit according the manufacturer’s instructions (Miltenyi Biotec, Bergisch Gladbach, Germany). For co-receptor expression, relative mean fluorescence intensity (MFI) was chosen to avoid any variability in antibody batches.

### Enrichment and culture of monocytes and CD4^+^ T cells

Monocytes from patients and healthy controls, and naïve CD4^+^ T cells from leukocyte depletion filters (CompoFlow) from healthy blood donors, were isolated using magnetic cell sorting (MACS, Monocyte isolation kit II and Naïve CD4^+^ cell isolation kit II, Miltenyi Biotec) as previously described [29, 30]. Purity of CD14^+^ monocytes was assessed to ≥85% for healthy controls and breast cancer patients, and ≥80% for sepsis patients using flow cytometry. The monocytes were immediately used for functional analyzes or frozen in TRIZOL (Invitrogen, Carlsbad, CA, USA) at -80°C for subsequent gene expression microarray.

### Quantitative RT-PCR

Total RNA from monocytes was isolated using TRIZOL. cDNA synthesis was performed using random hexamers and the M-MuLV reverse transcriptase enzyme (Thermo Scientific). Quantitative RT-PCR was performed in triplicates according to the manufacturer’s instructions using Maxima SYBR Green/Rox (Thermo Scientific). The relative *ARG1* mRNA expression was normalized to *ACTB*, *GAPDH* and *SDHA* and calculated using the comparative Ct method [31]. Primers used: ACTB forward; CTGGAACGGTGAAGGTGACA, ACTB reverse; AAGGGACTTCCTGTAACAATGCA, GAPDH forward; TGCACCACCAACTGCTTAGC, GAPDH reverse; GGCATGGACTGTGGTCATGAG, SDHA forward; TGGGAACAAGAGGGCATCTG, SDHA reverse; CCACCACTGCATCAAATTCATG, ARG1 forward; CAAGGTGGCAGAAGTCAAGAA, ARG1 reverse; GCTTCCAATTGCCAAACTGT.

### T cell suppression assay

0, 500, 5000 or 50 000 monocytes were co-cultured with 50 000 allogeneic naïve CD4^+^ T cells from healthy blood donors at indicated stimulator:responder ratios in OptiMEM (Gibco Life Technologies, Paisley, UK) supplemented with penicillin/streptomycin (Thermo Scientific, South Logan, Utah, USA), 10 ng/mL rhGM-CSF in all cultures and controls (added in order to improve cell survival as OptiMEM is nutrient-poor, R&D Systems, Minneapolis, MN, USA) and CD3/CD28 T cell activating dynabeads according to the manufacturer’s instructions (Gibco Life Technologies, AS, Oslo, Norway) for a total of 48h. 1 μCi [methyl-^3^H]thymidine was added for the last 18h and incorporation was measured in a Microbeta Counter (PerkinElmer, Boston, MA, USA). The background signal from monocytes was subtracted before calculating the relative proliferation of CD4^+^ T lymphocytes.

### Cytokine production

Monocytes were cultured in OptiMEM w/wo 100 ng/mL LPS (lipopolysaccharide, γ-irradiated from *Salmonella enterica* serotype typhimurium, #L6143 Sigma Aldrich, St. Louis, MO, USA) for 24h *ex vivo*. The production of IL-10, IL-12, IL-6, IL-1β, TNF, IL-8 and TGFβ was measured using Human Inflammatory Cytokine Cytometric Bead Array (CBA, BD Biosciences, San Diego, CA, USA) or Human TGFβ ELISA (R&D Systems) according to the manufacturers’ instructions. IL-12 was undetectable and hence excluded in this study.

### Gene expression analysis

Total RNA from monocytes (ca. 1x10^6^ cells) was isolated using TRIZOL. The integrity of the obtained RNA was assessed using Agilent 2100 Bioanalyzer (Agilent, Santa Clara, CA, USA). Samples were hybridized to Human HT-12 v4.0 Expression BeadChips (Illumina Inc, San Diego, CA, USA) at the SCIBLU Genomics Center at Lund University. Data normalization using quantile normalization and filtering of low quality probes (Detection P-value >0.01), and a presence filter excluding probes lacking data in more than 2 out of 13 samples were performed using BioArray Software Environment (BASE) [32]. The subsequent steps were performed in Multi Experiment Viewer 4.6 [33]. The data was log2 transformed and probes that varied the most across experiments (5775 probes) were selected for further analysis. The gene expression data is available at Gene expression Omnibus (GEO) with accession number GSE65517. Significance analysis of microarrays (SAM, [34]) was performed to identify differentially expressed genes between the patient groups. Probes were median centered across samples. Pearson correlation distance and average linkage were used for hierarchical clustering. Gene ontology analysis was performed on the 343 genes differentially expressed between breast cancer/sepsis monocytes and healthy control monocytes (FDR<0.05) using the Database for Annotation, Visualization and Integrated Discovery (DAVID) [35, 36]. Gene set enrichment analysis (GSEA) was performed on ranked gene lists generated based on significance of differential expression (SAM) between breast cancer patients (positive phenotype) and healthy controls (negative phenotypes) [37]. The two gene sets analyzed were gene lists containing all genes with significant differential expression between CARS (immunosuppressive) and SIRS (pro-inflammatory) phases of sepsis, respectively, as described by Xu *et al* [38].

### Statistical analysis

Statistical analyzes performed on leukocyte populations in peripheral blood were performed using non-parametric Mann-Whitney Wilcoxon test (SPSS 20.0, SPSS Inc, Armork, NY, USA). For comparison of clinicopathological characteristics, χ^2^ was used for comparison of categorized variables and the Mann-Whitney U test for continuous variables. A p-value of < 0.05 was taken for significant. SAM was used to identify differentially expressed genes between healthy controls and breast cancer/sepsis monocytes. Statistics used in gene expression analyzes are described above. All other analyzes statistics by Student’s t-test unless otherwise stated.

## Results

### Characterization of leukocyte populations in peripheral blood of breast cancer patients

In order to study the leukocyte populations present in breast cancer patients of varying degrees of severity, freshly isolated peripheral blood mononuclear cells (PBMCs) from patients with primary (early) breast cancer, patients with advanced breast cancer (*i*.*e*. patients with locoregional recurrence or distant metastasis; LRR/MBC) as well healthy controls (HC) and patients with sepsis (*i*.*e*. a systemic inflammatory response) were analyzed using flow cytometry. Clinical information is available in S1 Table. The percentage of lymphocyte and dendritic cell (DC) populations are summarized in Table 1.

**Table 1:** Flow cytometric analysis of leukocyte populations in the peripheral blood of healthy controls (HC), patients with early breast cancer (BC), patients with locoregional recurrence or metastatic breast cancer (LRR/MBC) and patients with sepsis.

		HC	Early BC	LRR/MBC	Sepsis
	*Leukocyte population*	(median ± SEM) *n* = 13	(median ± SEM) *n* = 10	(median ± SEM) *n* = 23	(median ± SEM) *n* = 13
*Lymphocyte populations*	% CD3^+^ cells of PBMCs	34.9 ± 4.1	29.7 ± 2.7	20.4 ± 2.4**	23.1 ± 3.0*
	% CD8^+^ cells of CD3^+^ cells	36.9 ±3.2	26.9 ± 2.7	35.7 ± 2.2	23.9 ± 3.1*
	% CD4^+^ cells of CD3^+^ cells	54.4 ± 3.3	66.5 ± 4.9	60.7 ± 3.4	71.5 ± 4.0**
	CD4:CD8 T cell ratio	1.4 ± 0.4	2.5 ± 0.4*	1.8 ± 0.3	2.9 ± 0.6*
	% CD4^+^CD25^+^CD127^low/-^ cells of CD4^+^ cells ^a^	3.1 ± 0.4	4.0 ± 0.4	3.2 ± 0.5	4.3 ± 0.4
	% CD19^+^ cells of PBMCs	3.6 ± 0.5	3.7 ± 0.5	3.3 ± 1.3 ^b^	4.0 ± 0.8
	% CD56^+^CD3^-^ cells of PBMCs	5.9± 0.6	4.2 ± 0.5	2.5 ± 0.5**	1.9 ± 0.8**
	% CD56^+^CD3^+^ cells of PBMCs	1.6 ± 0.4	1.4 ± 0.7	0.8 ± 0.3*	0.5 ± 0.2**
	% CD56^+^CD3^+^ cells of CD3^+^ cells	7.2 ± 1.0	5.1 ± 2.5	3.9 ± 1.7	2.4 ± 1.0
*DC populations*	% MDC1 of PBMCs ^c^	0.4 ± 0.1	0.3 ± 0.1	0.2 ± 0.0*	0.3 ± 0.0*
	% MDC2 of PBMCs ^d^	0.1 ± 0.0	0.0 ± 0.0	0.0 ± 0.0**	0.1 ± 0.0
	% PDC of PBMCs ^e^	0.5 ± 0.1	0.6 ± 0.3	0.5 ± 0.2	0.5 ± 0.1

Values are median percentage (±SEM) of population of PBMCs, total CD3^+^ T cell population or total CD4^+^ T cell population. Ratio CD4:CD8 are calculated as percentage CD4^+^ cells / percentage CD8^+^ cells. Statistics by Mann-Whitney Wilcoxon test

* p < 0.05,

** p < 0.01.

^a^ CD127 was used as an alternative marker for Tregs according to Liu *et al* 2006 [55].

^b^ *n* = 17 for LRR/MBC CD19^+^ cells of PBMCs.

^c^ MDC1; CD14^-^CD19^-^CD1c/BDCA-1^+^

^d^ MDC2; CD14^-^CD19^-^CD141/BDCA-3^high^

^e^ PDC; CD14^-^CD19^-^CD303/BDCA-2^+^

**Fig 1:**
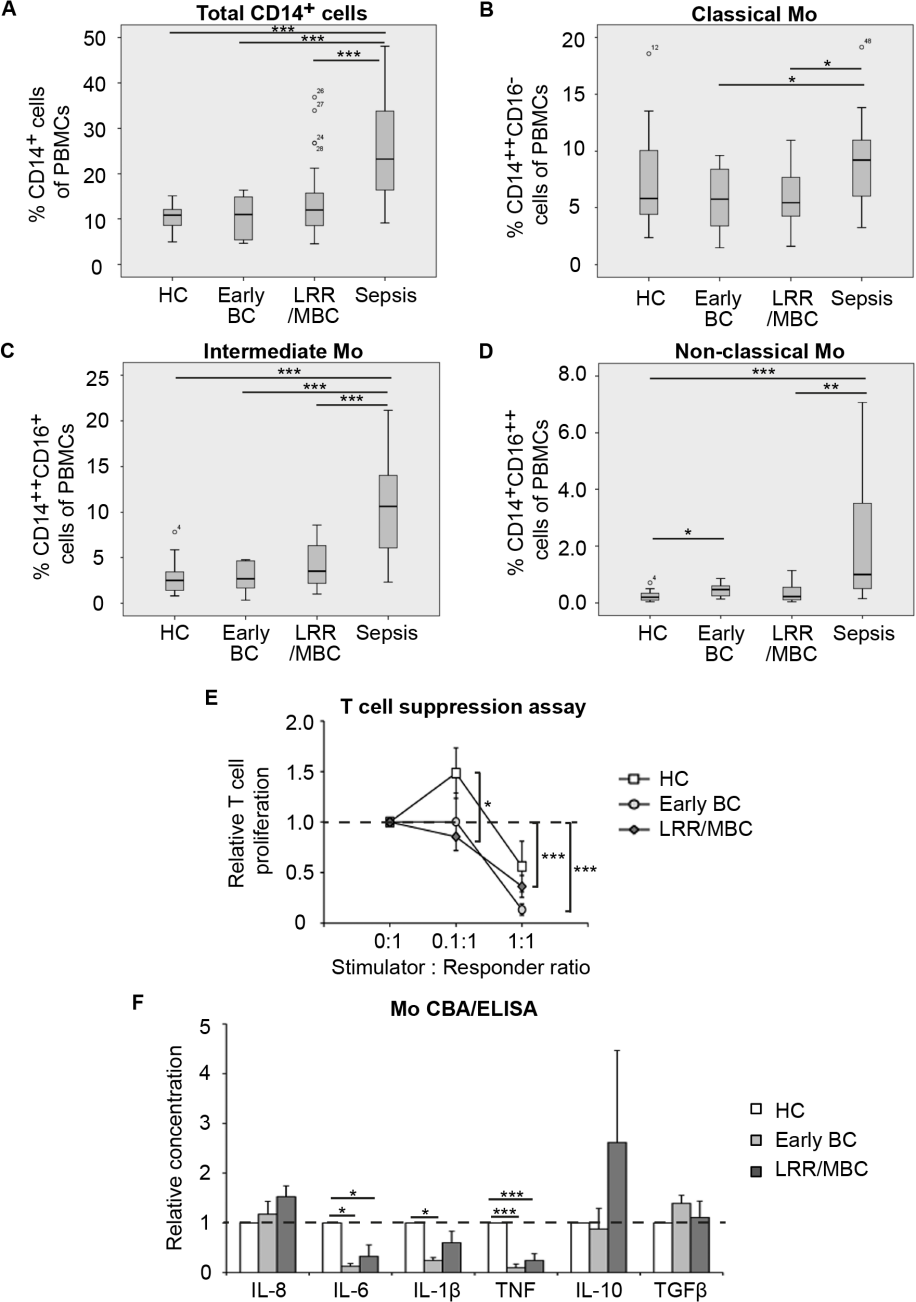
Monocytes from patients with advanced breast cancer display immunosuppressive properties. Flow cytometric analyzes of freshly isolated PBMCs from healthy controls (HC), patients with early breast cancer (BC), patients with advanced breast cancer (LRR/MBC) and patients with sepsis. (**A-D**) Total CD14^+^ monocytes (A), classical CD14^++^CD16^-^ monocytes (B), intermediate CD14^++^CD16^+^ monocytes (C) and non-classical CD14^+^CD16^++^ monocytes (D) of PBMCs. HC *n* = 13, Early BC *n* = 10, LRR/MBC *n* = 18 and sepsis *n* = 11. Statistics performed by Mann-Whitney Wilcoxon test, * < 0.05, ** p < 0.01, *** p < 0.001. (**E**) Monocytes (stimulators) were co-cultured with CD3/CD28 stimulated allogeneic naïve CD4^+^ T cells (responders) from healthy donors at indicated stimulator:responder ratios. T cell proliferation was assessed by thymidine incorporation. HC *n* = 13, Early BC *n* = 10 and LRR/MBC *n* = 11. Lines represent mean relative proliferation, bars; SEM. Statistics by Student’s t-test * p < 0.05, *** p < 0.001. (**F**) The spontaneous production of IL-8, IL-6, IL-1β, TNF and IL-10 from monocytes cultured *ex vivo* for 24h was analyzed using CBA. Mean concentrations from healthy control monocytes were put to 1. HC *n* = 10, Early BC *n* = 9 and LRR/MBC *n* = 16. The spontaneous production of TGFβ was analyzed using ELISA. HC *n* = 5, Early BC *n* = 5 and LRR/MBC *n* = 5. Statistics by one-way ANOVA. * p < 0.05, *** p < 0.001.

Circulating monocytes are key participants in innate immunity and frequently display altered phenotypes and functions in various diseases. However, all monocyte subpopulations investigated (total CD14^+^-, classical CD14^++^CD16^-^, intermediate CD14^++^CD16^+^- and non-classical CD14^+^CD16^++^ monocytes) were largely unaltered across breast cancer groups (Fig 1A–1D, see S1A Fig for representative dot plots). In accordance with previous studies, patients with sepsis displayed an elevated CD16^+^:CD16^-^ monocyte ratio (S1B Fig) [39]. This ratio was, however, only modestly elevated in patients with breast cancer (S1B Fig).

### Monocytes from breast cancer patients inhibit T cell proliferation and produce anti-inflammatory cytokines

In order to further elucidate the functional properties of monocytes from breast cancer patients, freshly isolated monocytes were co-cultured with CD3/CD28 activated naïve CD4^+^ T cells in an allogeneic T cell suppression assay. In line with what has previously been described, monocytes from healthy controls stimulate T cell proliferation at a stimulator:responder ratio of 0.1:1 (Fig 1E, corresponds to ratio of 1:8 or 0.1 in the cited references) [14, 15, 40]. This is followed by a suppression-slope, by both HC and patient monocytes, albeit with a stronger suppression induced by patient monocytes (Fig 1E) [14, 15, 40]. This may be attributed to the fact that also non-activated monocytes produce iNOS amongst other factors. The T cell suppression induced by monocytes prepared from LRR/MBC patients was significantly enhanced as compared to when induced by monocytes prepared from healthy controls at the ratio 0.1:1 (Fig 1E). Furthermore, monocytes from both LRR/MBC patients and from early breast cancer patients, suppressed T cell proliferation significantly at a stimulator:responder ratio of 1:1. This was in contrast to monocytes from healthy controls that induced a modest non-significant suppression (Fig 1E).

Next, we investigated the spontaneous production of IL-8, IL-6, IL-1β, TNF, IL-10 and TGFβ from monocytes cultured for 24h *ex vivo* using cytometric bead array (CBA) or enzyme-linked immunosorbent assay (ELISA). Monocytes from patients with LRR/MBC tended to secrete higher amounts of the pro-angiogenic and anti-inflammatory cytokines IL-8 and IL-10 when compared to healthy controls (Fig 1F). On the other hand, the secretion of IL-6, IL-1β and TNF was significantly lower in breast cancer patients (Fig 1F). Cytokine concentrations for each patient are depicted in S2 Fig TGFβ levels were unaltered. Interestingly, monocytes from patients with early breast cancer produced similar amounts of cytokines (IL-8, IL-6, IL-1β and TNF) as those observed in patients with advanced disease (LRR/MBC), suggesting that monocytes are functionally affected already early in the disease (Fig 1F). Furthermore, lipopolysaccharide (LPS) stimulated monocytes from breast cancer patients’ secreted similar quantities of cytokines when compared to healthy controls, confirming that the monocytes are indeed functional (S3A–S3F Fig, *left panels*). However, when comparing LPS stimulated versus untreated monocytes, the fold induction of cytokine secretion differed between breast cancer patients and healthy controls (S3A–S3F Fig, *right panels*). No difference regarding reactive oxygen species (ROS) production when compared to healthy controls was observed (data not shown).

### Patients with metastatic breast cancer exhibit an elevated Mo-MDSC population

**Fig 2:**
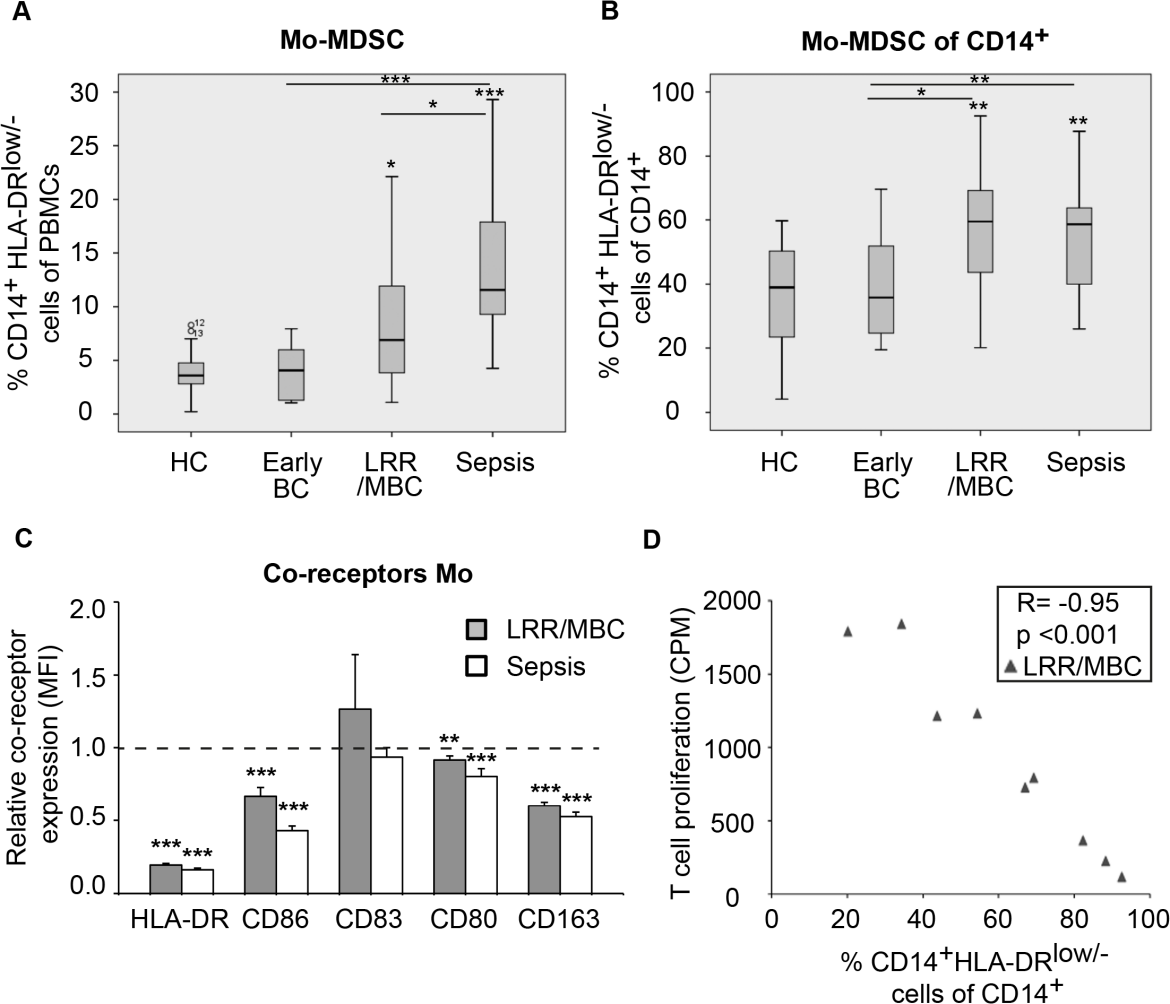
Increased frequency of Mo-MDSCs (CD14^+^HLA-DR^low/-^Co-receptor^low/-^) in patients with advanced breast cancer. Flow cytometric analyzes of freshly isolated PBMCs from healthy controls (HC), patients with early breast cancer (BC), patients with advanced breast cancer (LRR/MBC) and patients with sepsis. (**A**) The box plots represent the percentage of CD14^+^HLA-DR^low/-^ Mo-MDSC of PBMCs or (**B**) within total CD14^+^ monocyte population, HC *n* = 13, Early BC *n* = 10, LRR/MBC *n* = 25 and sepsis *n* = 18. Statistics were performed using Mann-Whitney Wilcoxon test. * p < 0.05, ** p < 0.01, *** p < 0.001. (**C**) Relative expression (mean fluorescence intensity; MFI) of co-receptors on CD14^+^HLA-DR^low/-^ monocytes from patients with LRR/MBC (grey) or sepsis (white) compared to CD14^+^HLA-DR^++^monocytes. Co-receptor expression on CD14^+^HLA-DR^++^ cells put to 1. Columns, mean; bars, SEM. Students t-test ** p < 0.01, *** p < 0.001. (**D**) T cell proliferation (CPM) *in vitro* at stimulator:responder ratio 0.01:1 correlates inversely with the percentage of Mo-MDSC within total CD14^+^ monocyte population. LRR/MBC *n* = 9, Spearman’s rho correlation.

The phenotype described above strongly resembles that of monocytic-MDSCs (Mo-MDSCs). Therefore, we analyzed the presence of CD14^+^HLA-DR^low/-^Co-receptor^low/-^ (i.e. negative for co-receptors such as CD86 and CD80) Mo-MDSCs in the peripheral blood of breast cancer patients. A gradual increase in Mo-MDSC of PBMCs was observed with disease progression (HC to early breast cancer to metastatic disease), with significantly elevated levels in LRR/MBC patients (Fig 2A). This increase was most prominent when examining Mo-MDSCs within the total CD14^+^ monocyte population (Fig 2B). The reduction in HLA-DR observed in breast cancer patients compared to healthy controls was not correlated to sex or age and was exclusive for monocytes as CD19^+^ B lymphocytes displayed normal or only slightly reduced levels of HLA-DR (data not shown). Interestingly, the surface phenotype of monocytes from breast cancer patients also displayed a striking similarity to the immunosuppressive monocytes seen in patients with sepsis. Traditionally, sepsis patients display increased proportions of reprogrammed CD14^+^HLA-DR^low/-^Co-receptor^low/-^ monocytes [25]. Indeed, we observed a significant enrichment of CD14^+^HLA-DR^low/-^ monocytes in the peripheral blood, as well as within the total monocyte pool, in patients with sepsis (Fig 2A–2B). In addition, breast cancer and sepsis HLA-DR^low/-^ monocytes, when compared to HLA-DR^++^ monocytes, displayed significantly reduced levels of the co-receptors CD86, CD80 and the suggested anti-inflammatory monocyte-macrophage marker CD163 (Fig 2C) [41]. However, we observed a slight increase in the expression of CD83 in HLA-DR^low/-^ monocytes from breast cancer patients (Fig 2C). This is in accordance with a recent study suggesting that melanoma Mo-MDSCs express high levels of CD83 [14]. Furthermore, and in line with previous studies in glioblastoma and transplant patients, the percentage of Mo-MDSCs within the total CD14^+^ monocyte population in LRR/MBC patients correlated inversely with *in vitro* T cell proliferation in the T cell suppression assay from Fig 1E (Fig 2D) [16, 40]. In accordance with this, an inverse correlation between Mo-MDSCs and CD3^+^ T cells with associated reduction in peripheral blood CD3^+^ T cells was observed (S4A–S4B Fig). A trend towards a positive correlation was seen between Mo-MDSCs and Treg in LRR/MBC patients (S4C Fig). Mean fluorescence intensity (MFI) of CD163 on HLA-DR^++^ and HLA-DR^low/-^ monocytes are shown in S5A Fig and representative dot plots of CD14, HLA-DR, CD86 and CD163 are depicted in S5B–S5D Fig.

### Altered monocyte co-receptor expression in a subpopulation of LRR/MBC patients

Although CD86 was expressed on virtually all CD14^+^ monocytes, the expression was substantially decreased in a subpopulation of patients with LRR/MBC as well as in patients with sepsis (S5C and S5E Fig). We have previously shown that breast cancer TAMs and peripheral blood monocytes from sepsis patients frequently display an increased expression of CD163 [29, 42]. In contrast, only six of 25 patients with LRR/MBC displayed a high frequency of CD163^+^ monocytes (S5D and S5F Fig). Interestingly, three of these six patients had normal levels of Mo-MDSCs, possibly indicating two different mechanisms for inducing immunosuppressive CD14^+^HLA-DR^low/-^ monocytes and anti-inflammatory CD163^+^ monocytes.

### The expansion of Mo-MDSCs correlates with disseminated disease

**Fig 3:**
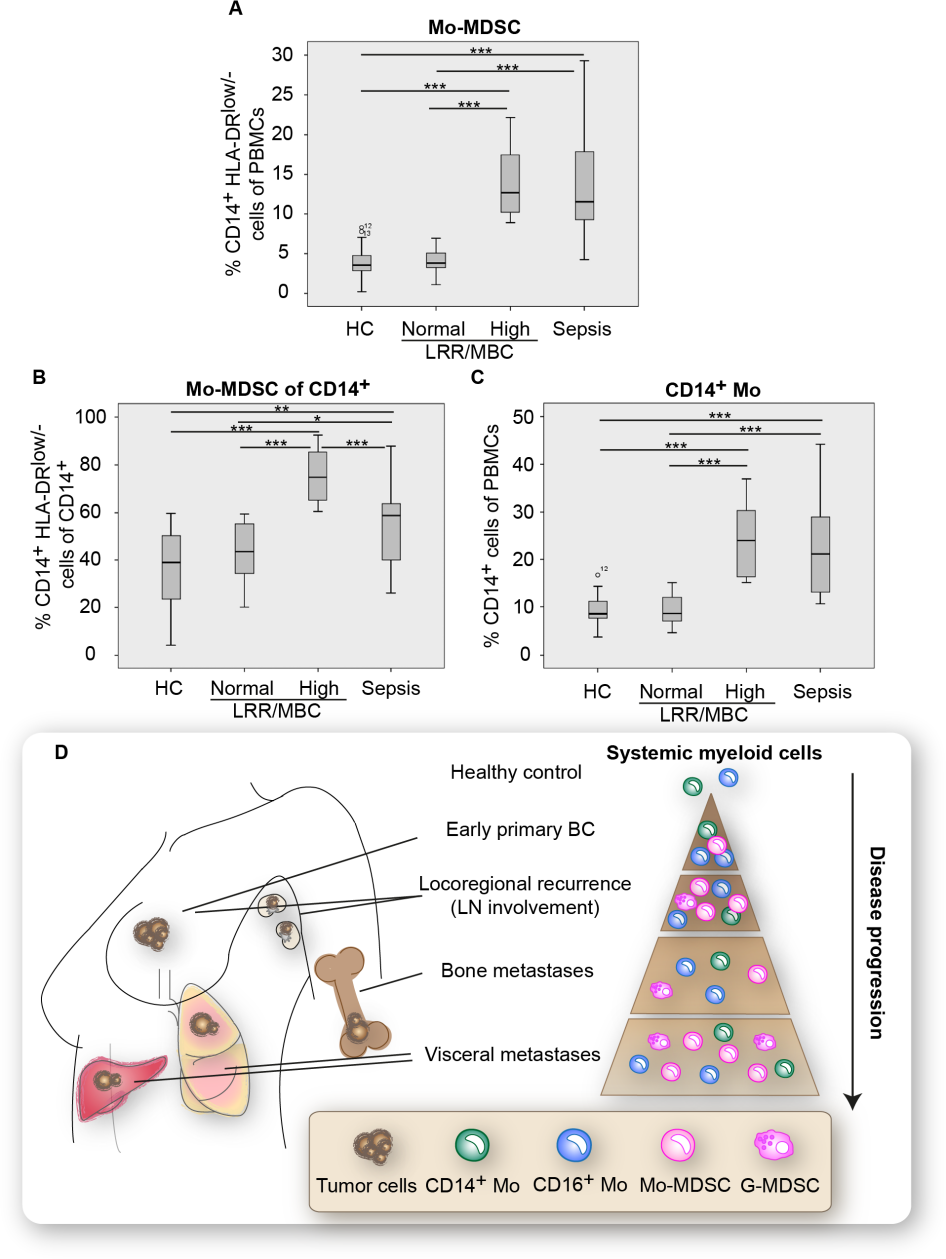
Mo-MDSCs are enriched in a subpopulation of patients with locoregional recurrence or metastatic breast cancer. (**A-C**) Flow cytometric analyzes of freshly isolated PBMCs from healthy controls (HC), patients with locoregional recurrence or metastatic breast cancer (LRR/MBC) or sepsis. The box plots represents the variation in respective cell population as percentage (%) of PBMCs. Cutoff into “normal” or “high” levels of Mo-MDSCs were based on the highest healthy control value. (A) Percentage of CD14+HLA-DRlow/- monocytes of PBMCs (HC *n* = 13, LRR/MBC “normal” *n* = 13, LRR/MBC “high” *n* = 12, Sepsis *n* = 18) or (B) within total CD14+ monocyte population (HC *n* = 13, LRR/MBC “normal” *n* = 13, LRR/MBC “high” *n* = 12, Sepsis *n* = 18). (C) Percentage of total CD14+ monocyte of PBMCs. HC *n* = 13, LRR/MBC “normal” *n* = 17, LRR/MBC “high” *n* = 8, Sepsis *n* = 18. (**D**) Cartoon presenting proposed frequencies of relevant systemic myeloid cell populations with disease progression. Abbreviations: Monocytes, Mo; Monocytic-MDSC, Mo-MDSC; granulocytic-MDSC, G-MDSC.

We observed that a subpopulation of patients with LRR/MBC displayed a substantial enrichment in Mo-MDSCs (S5B Fig). We therefore stratified the LRR/MBC patients into either “normal” (patients with Mo-MDSC levels comparable to healthy control) or “high” (patients with Mo-MDSC levels higher than the levels for healthy controls) (Fig 3A). The majority of patients in the “normal” group had locoregional or distant recurrence whereas the majority of patients in the “high” group presented with metastatic disease at initial diagnosis (Table 2). In addition, patients in the “high” group had more metastatic sites and significantly more patients had distant metastasis to lymph nodes (Table 2). Furthermore, patients with visceral organ metastases were overrepresented in the “high” group whereas patients with metastases restricted to the bone were typically found in the “normal” group (Table 2). Regarding ER status of the primary tumors, more ER negative tumors were found in the “high” group of patients. No differences were seen regarding age or previous adjuvant chemotherapy (Table 2). When this strategy was applied to the percentages of Mo-MDSC within CD14^+^ cells and total CD14^+^ monocytes of PBMCs (Fig 3B–3C), similar trends were observed. For proposed frequencies of myeloid populations with disease progression see Fig 3D. Altogether these results suggest that peripheral blood Mo-MDSCs correlate with disease progression in breast cancer and may represent a novel prognostic biomarker for breast cancer progression.

**Table 2:** Correlations between clinical parameters of patients with LRR/MBC and”normal” or”high” frequency of Mo-MDSCs of PBMCs (see Fig 3A).

		”Normal” Mo-MDSC	”High” Mo-MDSC	*P-value*
Mean age		62 ± 10 y	58 ± 10 y	0.3
**Tumor type**	Ductal	11	10	0.99
	Lobular	1	1	
	Other	1	1	
**NHG**	1	1	0	0.41
	2	5	4	
	3	5	1	
	Unknown	2	7	
**Tumor Size (T)**	T1	4	3	0.72
	T2	5	2	
	T3	1	2	
	T4	1	1	
	Unknown	2	4	
**Node status (N)**	N0	5	2	0.22
	N1	4	1	
	N2	2	4	
	Unknown	2	5	
**Hormone Receptor Status**	ER+	11	5	0.07
	ER-	2	5	
	Unknown	0	2	
	PR+	8	6	0.94
	PR-	5	4	
	Unknown	0	2	
**HER2 status**	HER2+	2	3	0.41
	HER2-	8	5	
	Unknown	3	4	
**Adjuvant chemotherapy**	Yes	4	3	0.75
	No	9	9	
**Type of recurrence**	Locoregional recurrence	2	0	***0*.*04***
	Distant recurrence	10	6	
	Distant metastasis at initial diagnosis	1	6	
**Number of metastatic sites**	0–2	9	4	0.07
	3–5	4	8	
**Metastatic site**	Lymph nodes	2	8	***0*.*009***
	Lung	4	4	0.9
	Liver	2	6	0.06
	Bone	9	11	0.16
	Visceral	5	9	0.07
	Bone-only	5	1	0.08
**Time to recurrence**		51 ± 50 m	52 ± 75 m	0.57

Using flow cytometry and functional assays, we noticed that the immune cell profile in patients with breast cancer was surprisingly similar to the immune cell profile observed in patients with sepsis (Fig 1, Table 1, Fig 3, S5, S6A and S6B Figs). In sepsis patients it has been suggested that granulocytic-MDSCs origin mainly from an emergency myelopoiesis [28]. Enrichment of Mo-MDSCs, on the other hand, may be due to either this or reprogramming of monocytes into an immunosuppressive state, or a combination of both mechanisms [23, 25]. Systemic signs of emergency myelopoiesis include increased release of immature cells from the bone marrow accompanied by leukocytosis and neutrophilia. However, in our material, only four out of twelve LRR/MBC patients with significantly increased Mo-MDSC levels displayed slightly elevated leukocyte and/or neutrophil counts (data not shown). The remaining eight LRR/MBC patients had normal leukocyte and neutrophil count, suggesting that emergency myelopoiesis is not the main cause of Mo-MDSC enrichment in these patients. This indicates that breast cancer Mo-MDSCs, similar to sepsis Mo-MDSCs, probably derive from already exported monocytes that become reprogrammed in the peripheral blood.

### Gene expression profiling of monocytes from breast cancer patients reveals similarity to re-programmed monocytes from sepsis patients

To address this question, we performed a gene expression microarray on isolated monocytes from patients with metastatic breast cancer, patients with sepsis as well as healthy controls. Total monocyte population including Mo-MDSCs, was chosen to enable comparison between healthy controls and patients. Metastatic breast cancer patients were chosen due to the significant enrichment of peripheral blood Mo-MDSCs in this group. In addition, monocytes from patients with tuberculosis were included as a control for a chronic local infection, as compared to the acute systemic inflammatory response (*i*.*e*. sepsis) and the inflammatory response during cancer. See S6C Fig for representative dot plots of monocytes from tuberculosis patients.

**Fig 4:**
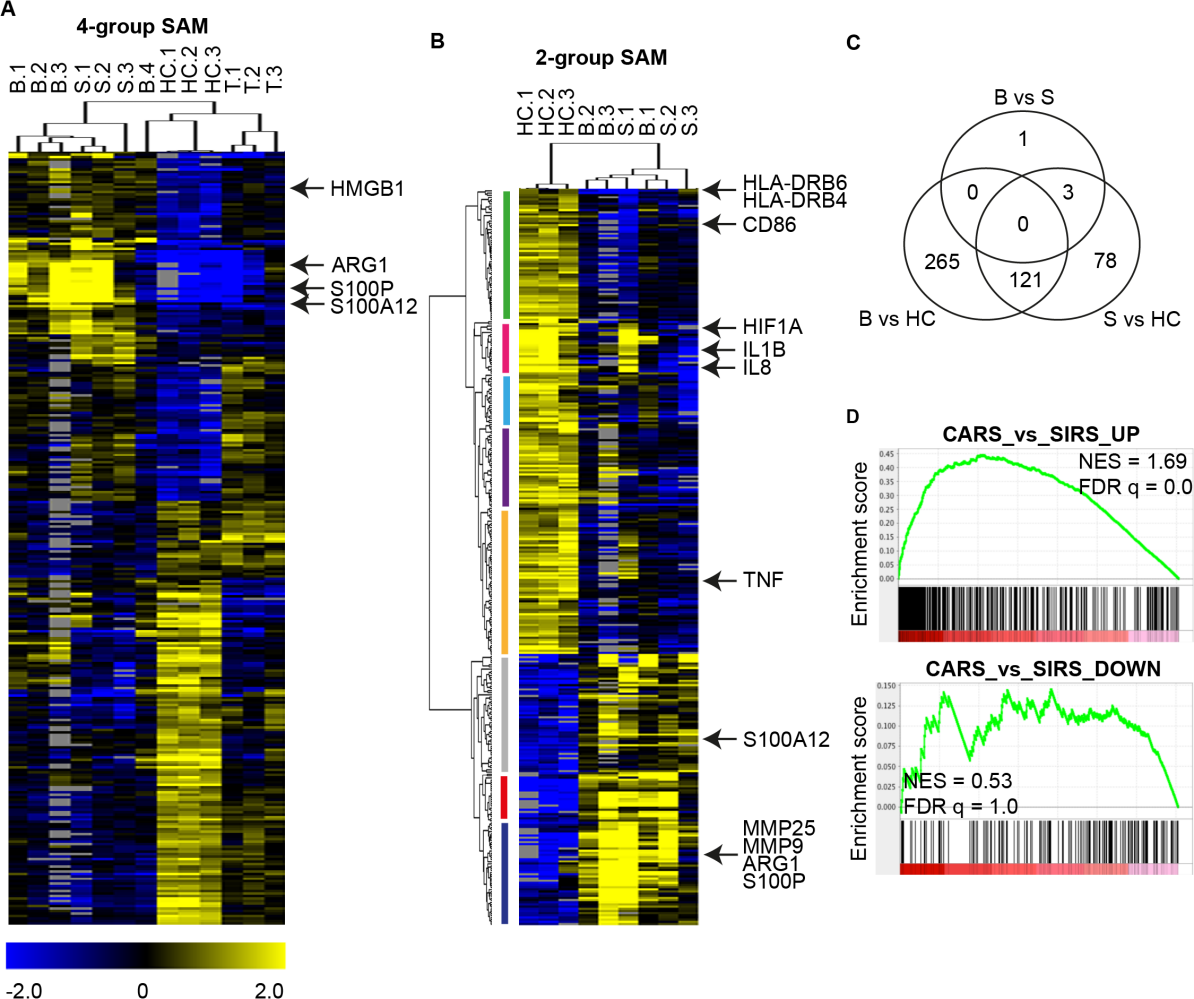
Gene expression profile analyzes show that monocytes from patients with metastatic breast cancer are similar to reprogrammed monocytes from sepsis patients. (**A**) Four-group significance analysis for microarray (SAM) between healthy controls (H.1-3), patients with metastatic breast cancer (MBC; B.1-4), sepsis (S.1-3) or tuberculosis (T.1-3) (312 genes, FDR<0.05), reveal that monocytes from MBC patients cluster with monocytes from sepsis patients. (**B)** Two-group SAM between monocytes from [MBC and sepsis patients] compared with monocytes from healthy controls (343 genes, FDR<0.05). Genes relevant in MDSCs or monocyte reprogramming are highlighted. See S3 and S4 Tables, and S1 File for detailed gene list. (**C**) Venn diagram of overlapping significant differentially expressed genes between monocytes from [patients with MBC (B) and HC (FDR <0.05)], [patients with sepsis (S) and HC (FDR < 0.05)] and [patients with MBC (B) and patients with sepsis (S; FDR < 0.25)]. Gene symbols are provided in S4 File. (**D**) Gene set enrichment analysis (GSEA) of CARS monocyte-associated (*top panel*) and SIRS monocyte-associated (*bottom panel*) gene sets [38] carried out on a gene list of all genes ranked according to their significance of differential expression between monocytes from MBCs (*n* = 3) versus HCs (*n* = 3). Normalized enrichment score (NES) and false discovery rate q-values are provided.

In order to look for expression pattern similarities amongst the patient samples, a four-group significance analysis for microarrays (SAM) and hierarchical clustering of significant differentially expressed genes (FDR<0.05, resulting in 312 genes) was performed. As shown in Fig 4A, two main clusters are apparent: 1) three of four breast cancer samples and all sepsis samples, 2) all healthy controls and tuberculosis samples, although at a separate branch within this cluster. This suggests similarities between monocytes in breast cancer and sepsis when compared with monocytes from healthy controls or tuberculosis patients. One patient with breast cancer, did however, cluster together with the healthy controls. This patient displayed a low/normal level of Mo-MDSCs and had metastases restricted to the bone whereas the remaining three breast cancer patients had elevated levels of Mo-MDSCs and also visceral organ metastases (S2 Table).

To identify differentially expressed genes between healthy controls and breast cancer/sepsis monocytes, we performed a two-group SAM between healthy controls and metastatic breast cancer/sepsis patients (excluding the breast cancer patient with low levels of Mo-MDSCs). The SAM analysis identified 343 differentially expressed genes (FDR<0.05) that separated the breast cancer/sepsis monocytes from the healthy controls (Fig 4B, S3 and S4 Tables). See S1 File for ranked gene list according to significance in the SAM analysis. Gene ontology was performed on genes with significantly higher and lower expression in breast cancer/sepsis monocytes compared to healthy control monocytes, respectively (S3 and S4 Tables and S2 and S3 Files). Several known MDSC-associated genes were highly up regulated in breast cancer/sepsis monocytes when compared with healthy controls, including e.g. ARG1 and S100A12 (Fig 4B and S4 Table) [11, 14, 43]. mRNA expression of the well described MDSC marker ARG1 in monocytes from healthy controls and patients are shown in S6D Fig. Breast cancer/sepsis monocytes also displayed higher expression of HMGB1 as well as several matrix metalloproteinases, which have previously been shown to be involved in the reprogramming of monocytes and metastatic/angiogenic processes respectively (Fig 4A–4B) [44–46]. In contrast, and in agreement with the established phenotype of reprogrammed monocytes as well as our cytokine and flow cytometry data (Fig 1E, S5 and S6B Figs), the expression of the pro-inflammatory cytokines TNF and IL-1β, as well as HLA-DR and CD86 was significantly higher in the healthy controls when compared to breast cancer/sepsis patients (Fig 4B) [25]. Among the 343 genes, 37 of were also differentially expressed when comparing breast cancer/sepsis monocytes to healthy control/tuberculosis monocytes (SAM, FDR<0.05, S4 Table). However, when specifically investigating the potential differences between breast cancer- and sepsis-derived monocytes using SAM analysis, only four genes were differentially expressed (FDR<0.25) between these two patient groups (Fig 4C and S4 File). On the other hand, a high proportion of the significant differentially expressed genes (FDR <0.05) were shared between metastatic breast cancer and sepsis when compared against healthy controls (Fig 4C and S4 File). Interestingly, the prototypical marker of MDSCs, ARG1, was amongst the shared genes, whereas HMGB1 is specific for monocytes derived from breast cancer patients (S4 File). Together this data reveals multiple similarities between peripheral blood monocytes derived from breast cancer and sepsis patients when compared to healthy controls.

### Breast cancer monocytes display enrichment of genes associated with monocytes from CARS patients

To further verify the similarities between breast cancer patients and sepsis patients as compared to healthy controls, gene set enrichment analysis (GSEA) was performed on ranked gene list of all genes according to the significance of their differential expression (SAM) between breast cancer patients and healthy controls. Two gene sets were obtained from Xu *et al* [38], which were generated based on significant differential expression between monocytes of CARS (*i*.*e* immunosuppressive phase which includes reprogrammed monocytes) and SIRS (*i*.*e*. acute pro-inflammatory) phases of sepsis patients. Interestingly, genes that were associated with CARS displayed a significant positive enrichment amongst genes that were higher expressed in breast cancer monocytes than in healthy controls (Fig 4D, *top panel*). On the other hand, genes associated with SIRS did not display any significant enrichment (Fig 4D, *bottom panel*). Taken together, these data support a similarity between monocytes of breast cancer and sepsis patients when compared to monocytes from other inflammatory-diseased (tuberculosis) patients or healthy controls. More specifically, expression profiles of monocytes from breast cancer patients displayed enrichment of genes associated with the CARS phase of sepsis, suggesting a stronger similarity between monocytes from breast cancer patients and the reprogrammed, immunosuppressive monocytes of sepsis patients.

## Discussion

Leukocytes have a paradoxical role in tumor progression. On one hand, leukocytes are involved in the physical destruction of neoplastic cells with the concomitant elimination of the tumor. On the other hand, leukocytes promote tumor growth, angiogenesis and metastasis [1, 2]. How breast cancer affects the systemic myeloid cell populations during disease progression as well as whether such alterations may be used to assess disease progression is largely unexplored.

In this study, we analyzed the peripheral blood leukocyte populations from patients with primary breast cancer, patients with locoregional recurrence as well as with distant metastasis in relation to healthy controls and sepsis patients. We noticed considerable alterations in the frequencies of several leukocyte populations in breast cancer patients. Notably, we found a significant enrichment of circulating Mo-MDSCs (CD14^+^HLA-DR^low/-^Co-receptor^low/-^) in patients with breast cancer. Although the Mo-MDSC surface phenotype was increasingly pronounced with disseminated disease, the monocytes from patients with early, primary, breast cancer were affected functionally. This suggests that already small, localized tumors induce a systemic response that affects circulating myeloid cells and is in agreement with gene expression studies on total peripheral blood cells from breast cancer patients showing that leukocytes are affected during early tumor development [47, 48]. Indeed, T cell proliferation was suppressed by monocytes from patients with early breast cancer as well as LRR/MBC. It is important to emphasize that in the T cell suppression assay used in this study, monocytes from LRR/MBC patients are not categorized into groups with normal or high levels of CD14^+^HLA-DR^low/-^ Mo-MDSCs. In fact, four of the thirteen LRR/MBC samples in the T cell suppression assay had high levels of Mo-MDSCs. Another drawback with the T cell suppression assay used in this study is that we chose to end at the ratio 1:1, whereas it normally would end at the ratio 0.5 (1:2) [14, 15, 40]. Regretfully, we do not have this ratio in our experiments. The Mo-MDSC levels did, however, correlate with increased T cell suppression. It may also be of interest to note that the higher ratio used for the T cell suppression assay may have limited relevance for the healthy controls as the monocyte / T cell ratio is lower in healthy controls compared to LRR/MBC patients (mean ratio of 0.37 and 0.81 for healthy controls and LRR/MBC patients, respectively).

Importantly, presence of Mo-MDSCs correlated with more severe disease, as patients with high frequency of Mo-MDSCs presented with more metastatic sites, lymph node- and visceral organ metastases. This is in accordance with previous studies where monocytes have been suggested to augment the invasive and metastatic potential of breast cancer cells [49–52]. Based on our findings, we suggest that monitoring of peripheral blood Mo-MDSCs may be a useful biomarker to assess disease progression in breast cancer patients as well as a possible therapeutic target. It is also interesting to note that six of the seven patients with disseminated disease at initial diagnosis had high levels of Mo-MDSCs. These patients still had the primary tumor in addition to the metastases when the blood was drawn. Furthermore, the patients with distant recurrence were distributed evenly across the “normal” and “high” Mo-MDSC groups. This may indicate that the primary tumor is more potent in inducing Mo-MDSCs than the metastases. Further studies are required in order to elucidate the impact that primary tumors and their respective metastases have on the Mo-MDSC population. Another explanation that cannot be ruled out is that breast cancer develops in immunosuppressed individuals as an opportunistic event. This is, however, outside the scope of this study, but is of interest to look into in the future.

The mechanism of Mo-MDSC generation is relatively unknown although tumor-derived factors (e.g. prostaglandins, growth factors, cytokines or pro-inflammatory factors such as S100A8/A9) may induce Mo-MDSC generation [11]. In addition, TLR-ligands (e.g. pathogen-associated molecular patterns; PAMPs or damage-associated molecular patterns; DAMPs, that occur in infectious and neoplastic disorders, respectively) are effector molecules known to affect MDSC generation [25]. In severe infections, such as sepsis, an initial rapid activation of innate immune cells occurs (SIRS), followed by an antagonistic anti-inflammatory and tissue regenerating response (CARS). This homeostatic response is partially mediated by a TLR-ligand and IL-10 dependent reprogramming of monocytes towards an immunosuppressive state (known as endotoxin tolerance), which results in an increased Mo-MDSC compartment [25]. Reprogramming of monocytes-macrophages during CARS has been extensively studied previously using gene expression profiling [38, 53]. When these gene expression profiles of monocytes derived from typical SIRS or CARS patients [38] were compared with our gene expression analyses, we found an enrichment of CARS associated genes amongst those genes that were upregulated in breast cancer monocytes. This may suggest that monocytes derived from breast cancer patients have gone through a reprogramming mechanism, resulting in an increased level of immunosuppressive Mo-MDSCs, as is observed in CARS patients. Tuberculosis, similar to cancer, is a chronic and local condition characterized by an anti-inflammatory response. Surprisingly, however, the gene expression profile of monocytes from patients with tuberculosis was very different from that of monocytes from breast cancer patients. This indicates that breast cancer- and sepsis-derived monocytes are indeed similar, rather than displaying a general state of activation. In sepsis patients, it has also been proposed that MDSCs may partly be generated by emergency myelopoiesis [23]. However, the vast majority of LRR/MBC patients used in this study displayed normal leukocyte and neutrophil counts. This suggests that breast cancer Mo-MDSCs are generated from monocytes in the blood by factors released from the tumor (e.g. anti-inflammatory cytokines or DAMPs) in a similar manner as during reprogramming in sepsis patients. This would be in line with the finding that also in patients with localized breast cancer, the monocytes display an immunosuppressive capacity, although the surface phenotype was yet unaltered. Indeed, this surface phenotype shift became significantly evident as the disease progressed. Interestingly, a recent publication displayed that DAMPs can induce tolerogenic macrophages in the same manner as PAMPs, supporting the findings presented here [54].

In this study we provide evidence for functionally immunosuppressive monocytes in early breast cancer, which gradually change surface phenotype towards typical Mo-MDSCs as the disease progresses. We also propose that cancer and sepsis induce not only similar immune responses but also alterations at a molecular level in circulating monocytes [24]. The similarities between monocytes derived from breast cancer patients and patients with severe sepsis (endotoxin tolerance reprogrammed monocytes; CD14^+^HLA-DR^low/-^), with regards to surface phenotype, functionality and gene expression profile, strongly suggests that monocytes in breast cancer patients are also being reprogrammed by similar mechanisms to those observed in sepsis patients, although with different TLR-ligands (e.g. DAMPs). Indeed, a classical DAMP, HMGB1 was enriched specifically in breast cancer derived monocytes. This could further imply that the typical Mo-MDSC surface phenotype is acquired late in this process. Due to the accessibility of peripheral blood Mo-MDSCs, it is possible that monitoring Mo-MDSC may be useful to assess disease progression and guide individualized immunomodulatory treatments of breast cancer patients. We suggest that in the future, however, targeted therapy towards myeloid immunosuppression could be considered already during early breast cancer disease and not only in patients with advanced disease.

## Supporting Information

S1 Fig Analyzes of monocyte subpopulations. (A-B) Peripheral blood mononuclear cells from healthy controls (HC), patients with early breast cancer (BC), locoregional recurrence or metastatic breast cancer (LRR/MBC) and sepsis were immediately stained for flow cytometry. The gates was set based on the CD14^-^CD16^-^ and CD14^++^CD16^-^ populations and according to Ziegler-Heitbrock *et al*, 2010 [56]. (A) Representative dot plots of CD14 and CD16. I) Non-classical CD14^+^CD16^++^ monocytes, II) intermediate CD14^++^CD16^+^ monocytes and III) classical CD14^++^CD16^-^ monocytes. (B) Ratio of CD16^+^ monocytes to CD16^-^ monocyte populations (percentage of CD14^+^CD16^+^ monocytes / percentage of CD14^+^CD16^-^ monocytes of PBMCs). HC *n* = 13, Early BC *n* = 10, LRR/MBC *n* = 18, Sepsis *n* = 10. Mann-Whitney Wilcoxon test * p < 0.05, ** p < 0.01.
(TIF)

S2 Fig Cytokine production by *ex vivo* cultured untreated monocytes. (A-E) The spontaneous production of indicated cytokines from monocytes cultured *ex vivo* for 24h were analyzed using cytometric bead array (CBA). Lines represent median concentration. HC *n* = 10, Early BC *n* = 9, LRR/MBC *n* = 16, Sepsis *n* = 8. (**F**) The spontaneous production of TGFβ from monocytes was analyzed using ELISA. Lines represent median concentration. HC *n* = 5, Early BC *n* = 5, LRR/MBC *n* = 5, Sepsis *n* = 5. Statistics performed by Mann-Whitney Wilcoxon test.
(TIF)

S3 Fig LPS-induced cytokine production by monocytes. (A-E, *left panels*) The production of indicated cytokines from LPS-stimulated monocytes were analyzed using CBA. Lines represent median concentration of respective cytokines. HC *n* = 10, Early BC *n* = 8, LRR/MBC *n* = 16, Sepsis *n* = 8. *Right panels*, columns represent mean fold induction of indicated cytokines in response to LPS; bars, SEM. (**F**, *left panel*) The TGFβ production from LPS-stimulated monocytes was analyzed using ELISA. Lines represent median cytokine concentration. HC *n* = 5, Early BC *n* = 4, LRR/MBC *n* = 5, Sepsis *n* = 5. *Right panel*, columns represent mean fold induction of indicated cytokines in response to LPS; bars, SEM. All statistics performed by Mann-Whitney Wilcoxon test * p < 0.05, ** p < 0.01.
(TIF)

S4 Fig Correlations between Mo-MDSCs and T cell populations. Flow cytometric analyzes of peripheral blood mononuclear cells (PBMCs) from healthy controls (HC), patients with early breast cancer (BC) and patients with locoregional recurrence or metastatic breast cancer (LRR/MBC). (**A**) An inverse correlation between percentage CD3^+^ T cells and percentage CD14^+^HLA-DR^low/-^ cells of total CD14^+^ cells was observed in breast cancer patients. Spearman correlation: HC *n* = 13 R = 0.14, Early BC *n* = 10 R = 0.13, LRR/MBC *n* = 22 R = -0.30. (**B**) Box plots represent the percentage of CD3^+^ T cells of PBMCs HC *n* = 13, Early BC *n* = 10, LRR/MBC *n* = 23. Statistics by Mann Whitney Wilcoxon test. * p < 0.05, ** p < 0.01. (**C**) A modest positive correlation between percentage of CD4^+^CD25^+^CD127^low/-^ Treg and percentage CD14^+^HLA-DR^low/-^ cells of total CD14^+^ cells in LRR/MBC patients. Spearman correlation: HC *n* = 13 R = 0.19, Early BC *n* = 10 R = -0.21, LRR/MBC *n* = 22 R = 0.30.
(TIF)

S5 Fig Gating strategies of monocytes. Peripheral blood mononuclear cells from healthy controls (HC), patients with early breast cancer (BC), locoregional recurrence or metastatic breast cancer (LRR/MBC) and sepsis were immediately stained for flow cytometry. (**A**) CD163 expression on HLA-DR^++^ and HLA-DR^low/-^ monocytes from patients with LRR/MBC. *Left panel;* Dot plot of MFI of CD163. *Right panel;* Representative histograms of CD163. (**B-D**) Representative dot plots of HLA-DR, CD86 and CD163 on CD14^+^ monocytes respectively. The numbers represent percentage in gate. The HLA-DR gate was set according to the invariant CD14^-^HLA-DR^++^ population and the CD163 gate based on the highest healthy control levels. (**E**) Box plot of percentage of CD14^+^CD86^+^ cells of CD14^+^ monocytes. HC *n* = 13, Early BC *n* = 10, LRR/MBC *n* = 25 and Sepsis *n* = 18. (**F**) Box plot of percentage of CD14^+^CD163^+^ cells of CD14^+^ monocytes. HC *n* = 13, Early BC *n* = 10, LRR/MBC *n* = 25 and Sepsis *n* = 18.
(TIF)

S6 Fig Functional assays of sepsis-derived monocytes. (**A**) T cell suppression assay as previously described. Lines represent mean relative proliferation at indicated stimulator:responder ratio; bars, SEM. HC *n* = 13, LRR/MBC *n* = 11, Sepsis *n* = 6. Statistics by Student’s t-test * p < 0.05, ** p < 0.01, *** p < 0.001. (**B**) The spontaneous production of IL-8, IL-6, IL-1β, TNF and IL-10 from monocytes cultured *ex vivo* for 24h was analyzed using CBA. Mean concentrations from HC monocytes were put to 1. HC *n* = 10, LRR/MBC *n* = 16, Sepsis *n* = 8. TGFβ production was analyzed using ELISA. HC *n* = 5 and LRR/MBC *n* = 5, Sepsis *n* = 5. Statistics by Mann-Whitney Wilcoxon test, * p < 0.05, ** p < 0.01, *** p < 0.001. (**C**) PBMCs from patients with tuberculosis (TB) were stained for flow cytometry. Representative dot plots of HLA-DR (*left panel*) or CD16 (*right panel*) on CD14^+^ monocytes are shown. (**D**) Quantitative RT-PCR analysis of ARG1 from monocytes. Mean relative mRNA expression from HC monocytes were put to 1. HC *n* = 4, Early BC *n* = 3, LRR/MBC *n* = 3, Sepsis *n* = 3. Statistics by Student’s t-test * p < 0.05.
(TIF)

S1 File SAM analysis between monocytes derived from patients with metastatic breast cancer and sepsis compared to monocytes derived from healthy controls, showing the 343 most differentially expressed genes between breast cancer/sepsis monocytes when compared with healthy control monocytes. Genes are ranked according to significance in the SAM analysis.
(XLS)

S2 File Gene ontology analysis performed on genes with significantly lower expression in [MBC / Sepsis] as compared to HC (FDR < 0.05; see S3 Table) using the Database for Annotation, Visualization and Integrated Discovery (DAVID). Significance was determined using two-group significance analysis of microarrays (SAM), FDR < 0.05, between monocytes from patients with metastatic breast cancer (MBC) and sepsis [MBC / Sepsis] compared to monocytes from healthy controls [HC] (excluding tuberculosis patients and the breast cancer patient that clustered with healthy controls).
(XLS)

S3 File Gene ontology analysis performed on genes with significantly higher expression in [MBC / Sepsis] as compared to HC (FDR < 0.05; see S4 Table) using the Database for Annotation, Visualization and Integrated Discovery (DAVID). Significance was determined using two-group significance analysis of microarrays (SAM), FDR < 0.05, between monocytes from patients with metastatic breast cancer (MBC) and sepsis [MBC / Sepsis] compared to monocytes from healthy controls [HC] (excluding tuberculosis patients and the breast cancer patient that clustered with healthy controls).
(XLS)

S4 File List of genes corresponding to the Venn diagram (Fig 4C). Abbreviations: HC; healthy controls, B; metastatic breast cancer, S; sepsis.
(XLS)

S1 Table Clinical characteristics of the breast cancer patients studied. Hormone receptor negative characterized as < 10% positive cells. T1 <20mm, T2 21–50mm, T3 >50mm, T4 growing into chest wall or skin.
(PDF)

S2 Table Clinical characteristics of patients with metastatic breast cancer (MBC) included in the microarray analyses. Abbreviations: distant metastases at time of initial diagnosis (MetDiag) and distant recurrence (DR).
(PDF)

S3 Table Genes with lower expression in breast cancer/sepsis-derived monocytes compared to monocytes from healthy controls. Two-group significance analysis of microarrays (SAM) between monocytes from patients with metastatic breast cancer (MBC) and sepsis [MBC / Sepsis] compared to monocytes from healthy controls [HC] (excluding tuberculosis patients and the breast cancer patient that clustered with healthy controls). The table specifies the 217 genes with significantly lower expression in [MBC / Sepsis] as compared to HC (FDR < 0.05) and relevant pathways as identified by gene ontology (DAVID). Genes of special interest in MDSCs and monocyte reprogramming are highlighted in red.
(PDF)

S4 Table Genes with higher expression in breast cancer/sepsis-derived monocytes compared to monocytes from healthy controls. Two-group significance analysis of microarrays (SAM) between monocytes from patients with metastatic breast cancer (MBC) and sepsis [MBC / Sepsis] compared to monocytes from healthy controls [HC] (excluding tuberculosis patients and the breast cancer patient that clustered with healthy controls). The table specifies the 126 genes with significantly higher expression in [MBC / sepsis] as compared to HC (FDR < 0.05) and relevant pathways as identified by gene ontology (DAVID). Genes in bold are the 37 significantly differentially expressed genes (SAM, FDR<0.05) between 1) monocytes from patients with [MBC / sepsis] and 2) monocytes from [HC / tuberculosis]. Genes of special interest in MDSCs and monocyte reprogramming are highlighted in red.
(PDF)
