# Supplementary figures and images for: Systemic Monocytic-MDSCs Are Generated from Monocytes and Correlate with Disease Progression in Breast Cancer Patients
Source: PLoS One. 2015 May 20;10(5):e0127028. doi: 10.1371/journal.pone.0127028 (PMC4439153; doi:10.1371/journal.pone.0127028)

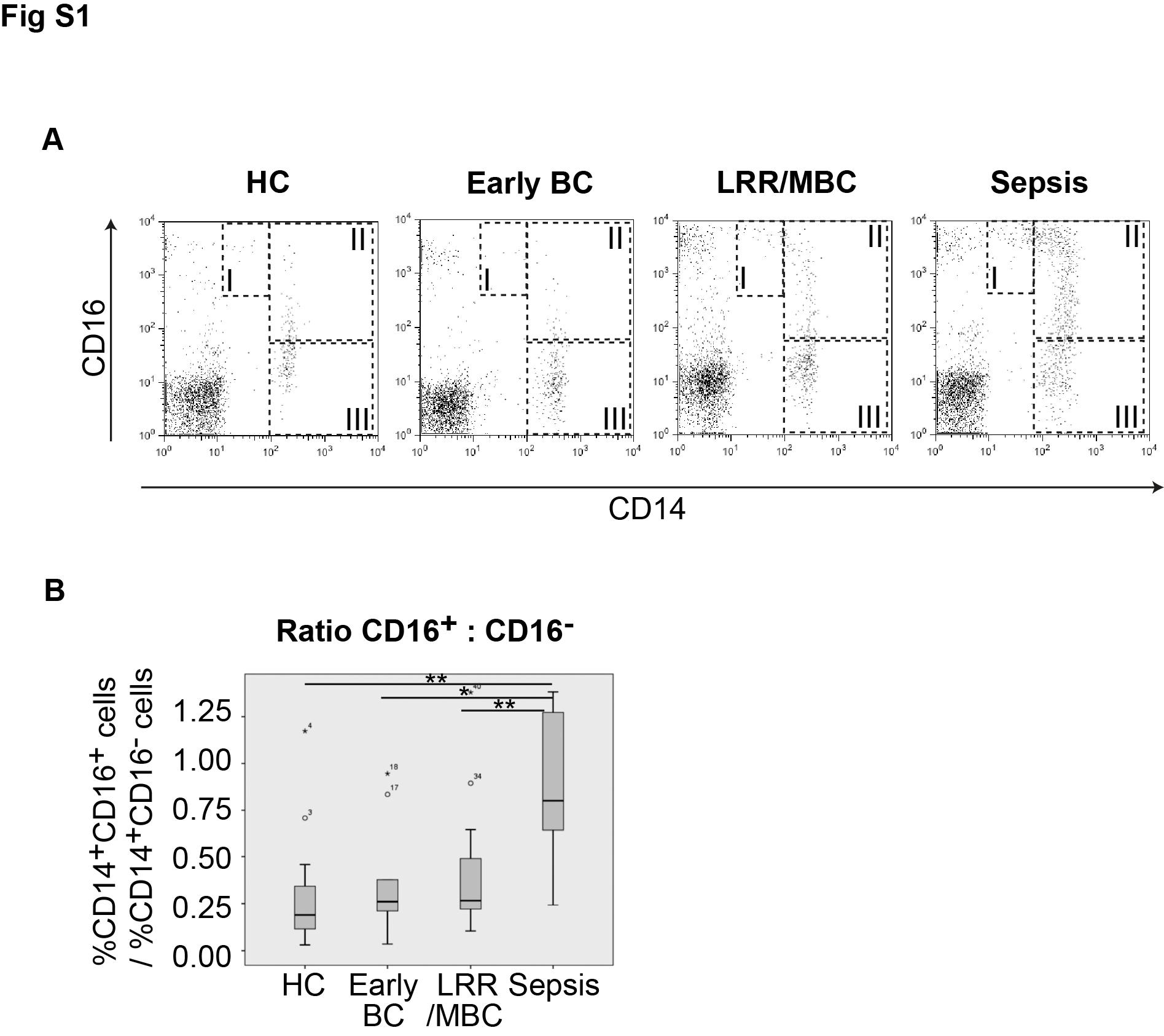

Supplement: S1 Fig — (A-B) Peripheral blood mononuclear cells from healthy controls (HC), patients with early breast cancer (BC), locoregional recurrence or metastatic breast cancer (LRR/MBC) and sepsis were immediately stained for flow cytometry. The gates was set based on the CD14-CD16- and CD14++CD16- populations and according to Ziegler-Heitbrock et al, 2010 [56]. (A) Representative dot plots of CD14 and CD16. I) Non-classical CD14+CD16++ monocytes, II) intermediate CD14++CD16+ monocytes and III) classical CD14++CD16- monocytes. (B) Ratio of CD16+ monocytes to CD16- monocyte populations (percentage of CD14+CD16+ monocytes / percentage of CD14+CD16- monocytes of PBMCs). HC n = 13, Early BC n = 10, LRR/MBC n = 18, Sepsis n = 10. Mann-Whitney Wilcoxon test * p < 0.05, ** p < 0.01. (TIF) [file pone.0127028.s001.tif]

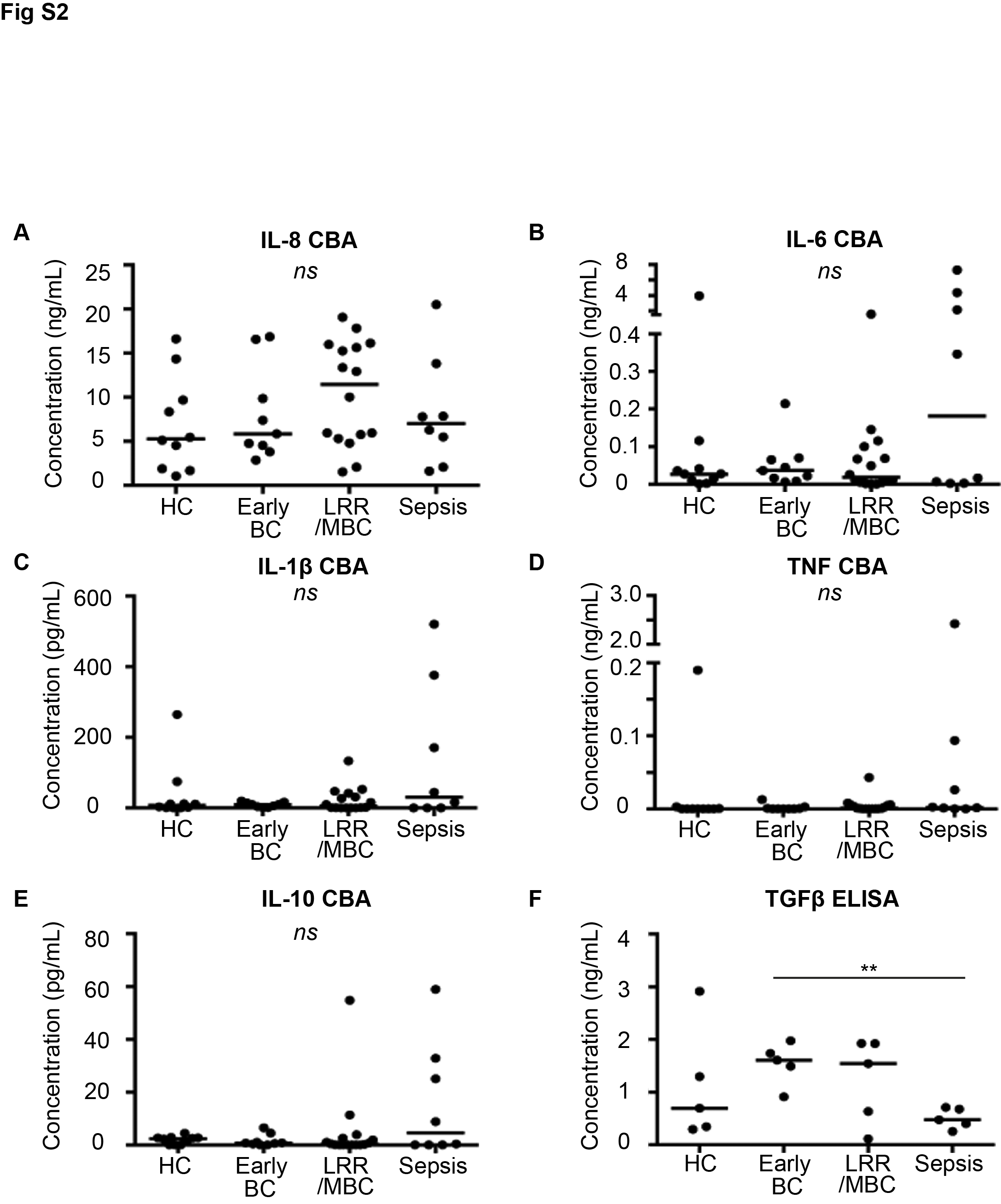

Supplement: S2 Fig — (A-E) The spontaneous production of indicated cytokines from monocytes cultured ex vivo for 24h were analyzed using cytometric bead array (CBA). Lines represent median concentration. HC n = 10, Early BC n = 9, LRR/MBC n = 16, Sepsis n = 8. (F) The spontaneous production of TGFβ from monocytes was analyzed using ELISA. Lines represent median concentration. HC n = 5, Early BC n = 5, LRR/MBC n = 5, Sepsis n = 5. Statistics performed by Mann-Whitney Wilcoxon test. (TIF) [file pone.0127028.s002.tif]

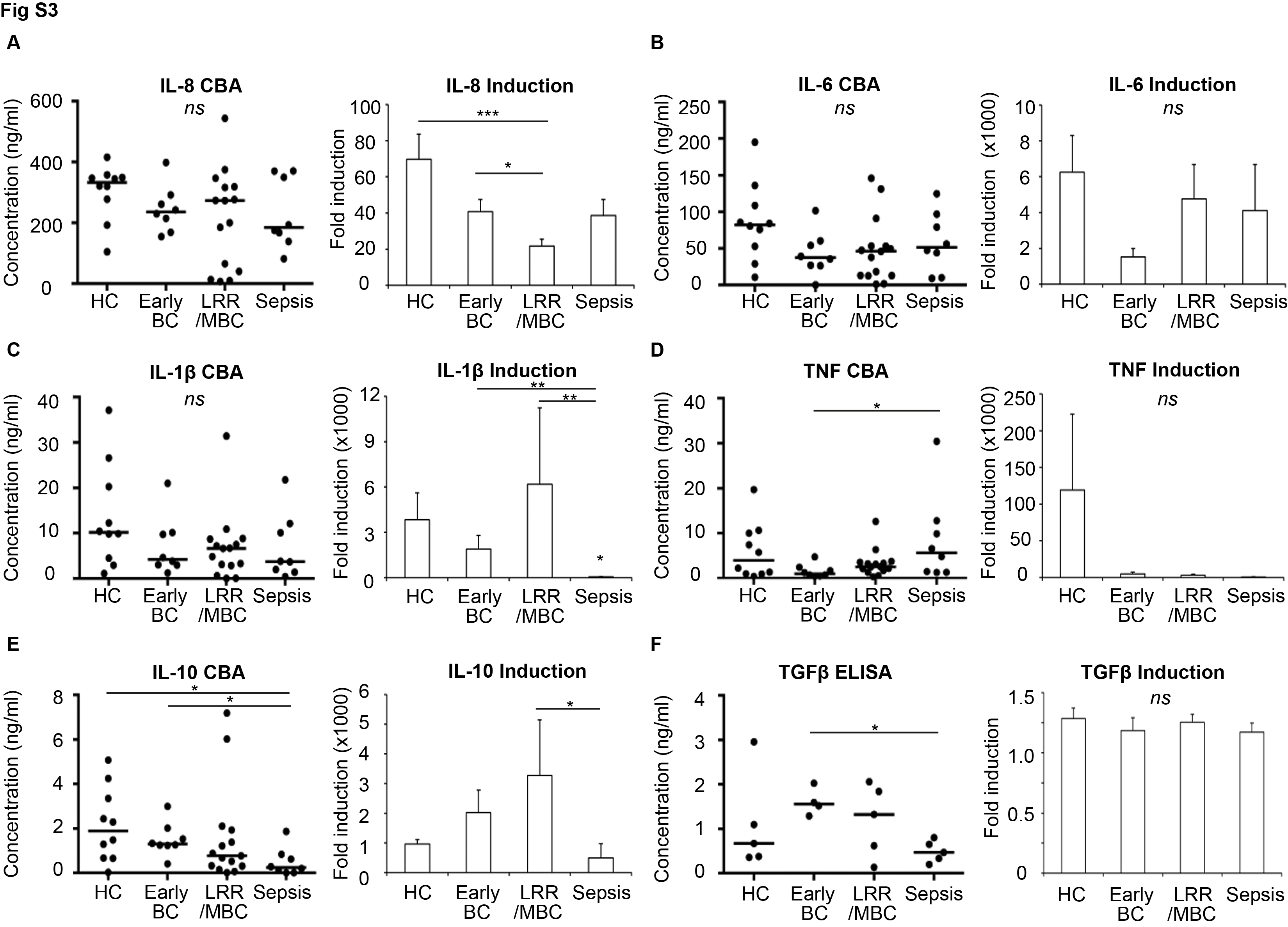

Supplement: S3 Fig — (A-E, left panels) The production of indicated cytokines from LPS-stimulated monocytes were analyzed using CBA. Lines represent median concentration of respective cytokines. HC n = 10, Early BC n = 8, LRR/MBC n = 16, Sepsis n = 8. Right panels, columns represent mean fold induction of indicated cytokines in response to LPS; bars, SEM. (F, left panel) The TGFβ production from LPS-stimulated monocytes was analyzed using ELISA. Lines represent median cytokine concentration. HC n = 5, Early BC n = 4, LRR/MBC n = 5, Sepsis n = 5. Right panel, columns represent mean fold induction of indicated cytokines in response to LPS; bars, SEM. All statistics performed by Mann-Whitney Wilcoxon test * p < 0.05, ** p < 0.01. (TIF) [file pone.0127028.s003.tif]

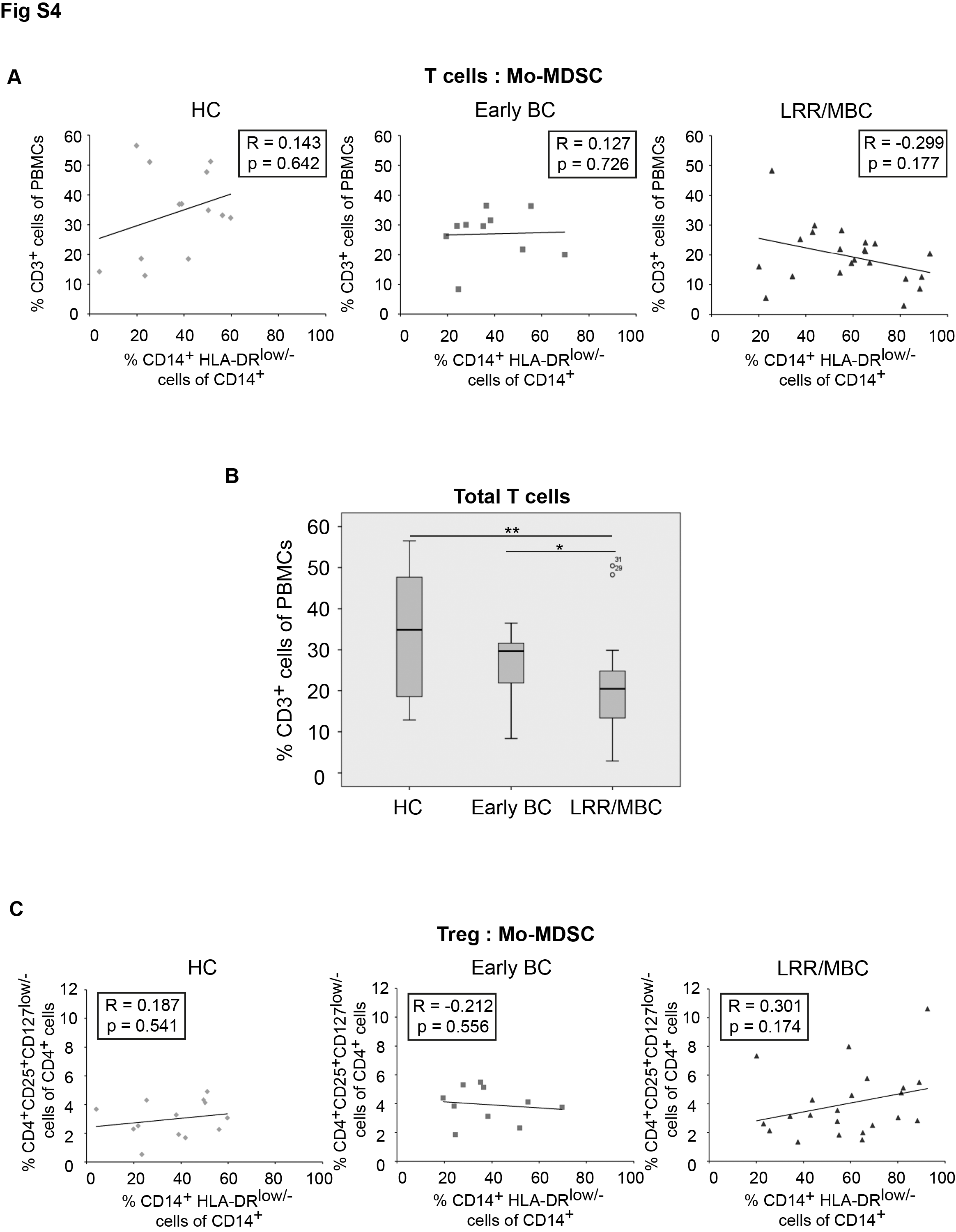

Supplement: S4 Fig — Flow cytometric analyzes of peripheral blood mononuclear cells (PBMCs) from healthy controls (HC), patients with early breast cancer (BC) and patients with locoregional recurrence or metastatic breast cancer (LRR/MBC). (A) An inverse correlation between percentage CD3+ T cells and percentage CD14+HLA-DRlow/- cells of total CD14+ cells was observed in breast cancer patients. Spearman correlation: HC n = 13 R = 0.14, Early BC n = 10 R = 0.13, LRR/MBC n = 22 R = -0.30. (B) Box plots represent the percentage of CD3+ T cells of PBMCs HC n = 13, Early BC n = 10, LRR/MBC n = 23. Statistics by Mann Whitney Wilcoxon test. * p < 0.05, ** p < 0.01. (C) A modest positive correlation between percentage of CD4+CD25+CD127low/- Treg and percentage CD14+HLA-DRlow/- cells of total CD14+ cells in LRR/MBC patients. Spearman correlation: HC n = 13 R = 0.19, Early BC n = 10 R = -0.21, LRR/MBC n = 22 R = 0.30. (TIF) [file pone.0127028.s004.tif]

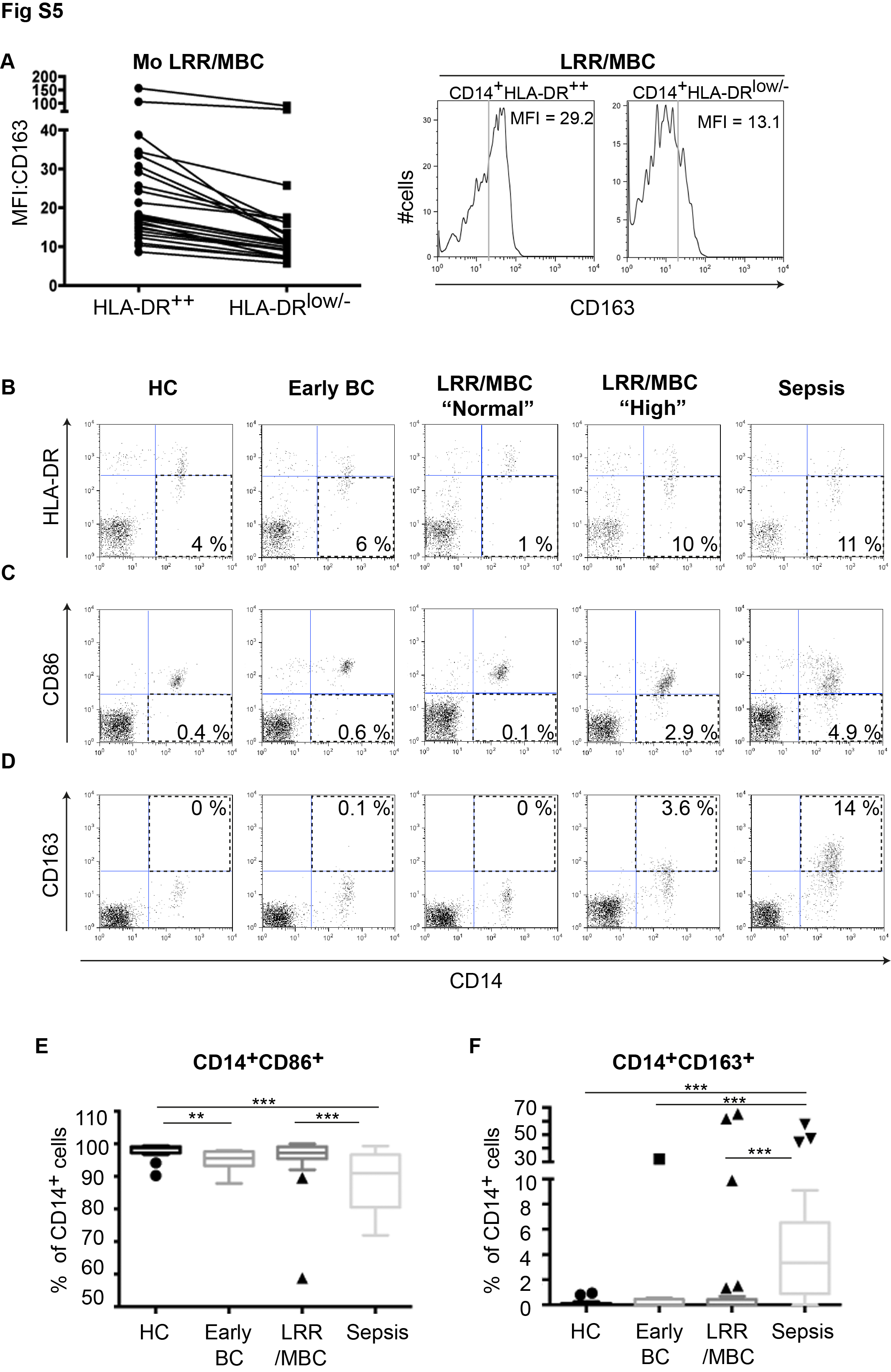

Supplement: S5 Fig — Peripheral blood mononuclear cells from healthy controls (HC), patients with early breast cancer (BC), locoregional recurrence or metastatic breast cancer (LRR/MBC) and sepsis were immediately stained for flow cytometry. (A) CD163 expression on HLA-DR++ and HLA-DRlow/- monocytes from patients with LRR/MBC. Left panel; Dot plot of MFI of CD163. Right panel; Representative histograms of CD163. (B-D) Representative dot plots of HLA-DR, CD86 and CD163 on CD14+ monocytes respectively. The numbers represent percentage in gate. The HLA-DR gate was set according to the invariant CD14-HLA-DR++ population and the CD163 gate based on the highest healthy control levels. (E) Box plot of percentage of CD14+CD86+ cells of CD14+ monocytes. HC n = 13, Early BC n = 10, LRR/MBC n = 25 and Sepsis n = 18. (F) Box plot of percentage of CD14+CD163+ cells of CD14+ monocytes. HC n = 13, Early BC n = 10, LRR/MBC n = 25 and Sepsis n = 18. (TIF) [file pone.0127028.s005.tif]

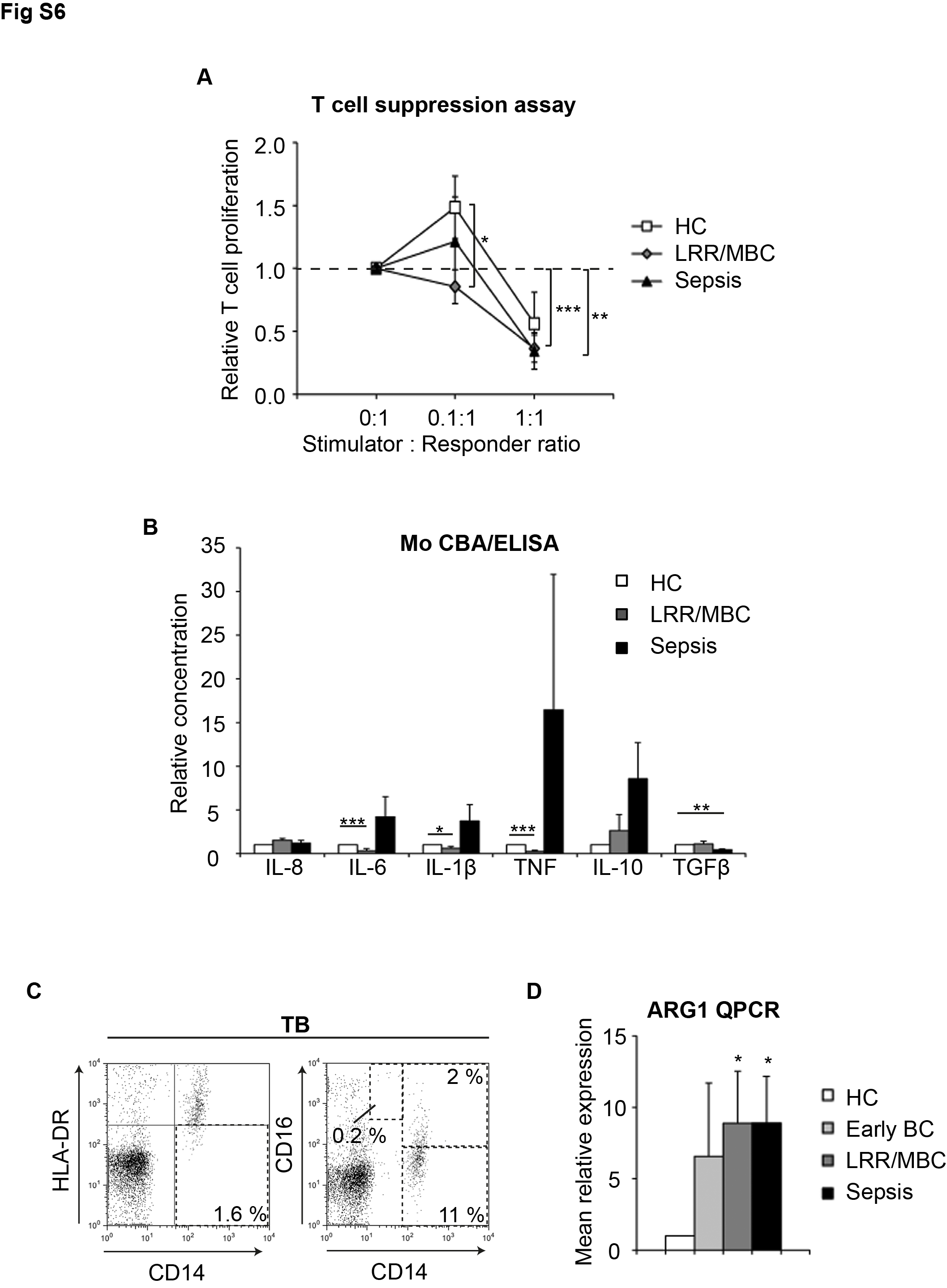

Supplement: S6 Fig — (A) T cell suppression assay as previously described. Lines represent mean relative proliferation at indicated stimulator:responder ratio; bars, SEM. HC n = 13, LRR/MBC n = 11, Sepsis n = 6. Statistics by Student’s t-test * p < 0.05, ** p < 0.01, *** p < 0.001. (B) The spontaneous production of IL-8, IL-6, IL-1β, TNF and IL-10 from monocytes cultured ex vivo for 24h was analyzed using CBA. Mean concentrations from HC monocytes were put to 1. HC n = 10, LRR/MBC n = 16, Sepsis n = 8. TGFβ production was analyzed using ELISA. HC n = 5 and LRR/MBC n = 5, Sepsis n = 5. Statistics by Mann-Whitney Wilcoxon test, * p < 0.05, ** p < 0.01, *** p < 0.001. (C) PBMCs from patients with tuberculosis (TB) were stained for flow cytometry. Representative dot plots of HLA-DR (left panel) or CD16 (right panel) on CD14+ monocytes are shown. (D) Quantitative RT-PCR analysis of ARG1 from monocytes. Mean relative mRNA expression from HC monocytes were put to 1. HC n = 4, Early BC n = 3, LRR/MBC n = 3, Sepsis n = 3. Statistics by Student’s t-test * p < 0.05. (TIF) [file pone.0127028.s006.tif]
