# Supplementary material for: Systemic Monocytic-MDSCs Are Generated from Monocytes and Correlate with Disease Progression in Breast Cancer Patients
Source: PLoS One. 2015 May 20;10(5):e0127028. doi: 10.1371/journal.pone.0127028 (PMC4439153; doi:10.1371/journal.pone.0127028)
Supplement: S1 Table — Hormone receptor negative characterized as < 10% positive cells. T1 <20mm, T2 21–50mm, T3 >50mm, T4 growing into chest wall or skin. (PDF) [file pone.0127028.s011.pdf]

Table S1. Clinical characteristics of the breast cancer patients studied. Hormone receptor negative characterized as < 10 % positive cells. T1 <20mm, T2 21-50mm, T3 >50mm, T4 growing into chest wall or skin.

	**Patients with early, primary, breast cancer** *n* = 10	**Patients with locoregional recurrence or metastatic breast cancer** *n* = 25
**Mean age** (years ± SD)	61 ± 8	60 ± 10
**Tumor type**		
Ductal	6	20
Lobular	2	3
Unknown	2	2
**Hormone receptor status**		
ER positive	8	16
ER negative	1	7
ER unknown	1	2
PR positive	5	14
PR negative	4	9
PR unknown	1	2
HER2 amplified	1	5
HER2 normal	9	13
HER2 unknown	1	7
**Tumor grade** (NHG)		
I	1	1
II	1	9
III	3	6
unknown	5	9
**Tumor size**		
T1	4	7
T2	5	7
T3	0	3
T4	0	2
unknown	1	6
**Lymph node status**		
Negative	3	7
1-3 positive nodes	5	5
>/= 4 positive nodes	1	6
unknown	1	7
**Distant metastasis at initial diagnosis**	0	7
