# Supplementary material for: Systemic Monocytic-MDSCs Are Generated from Monocytes and Correlate with Disease Progression in Breast Cancer Patients
Source: PLoS One. 2015 May 20;10(5):e0127028. doi: 10.1371/journal.pone.0127028 (PMC4439153; doi:10.1371/journal.pone.0127028)
Supplement: S2 Table — Abbreviations: distant metastases at time of initial diagnosis (MetDiag) and distant recurrence (DR). (PDF) [file pone.0127028.s012.pdf]

Table S2. Clinical characteristics of patients with metastatic breast cancer (MBC) included in the microarray analyses. Abbreviations: distant metastases at time of initial diagnosis (MetDiag) and distant recurrence (DR).

**Patient**	**Age** (years)	**High/low Mo-MDSC of PBMCs**	**Diagnosis**	**Adjuvant chemotherapy**	**Time to recurrence** (months)	**Metastases**
B.1	64	High	MetDiag	No	0	Visceral
B.2	71	High	DR	No	228	Visceral
B.3	52	High	DR	Yes	14	Visceral
B.4	50	Low	DR	Yes	56	Bone-only
