# Supplementary material for: Systemic Monocytic-MDSCs Are Generated from Monocytes and Correlate with Disease Progression in Breast Cancer Patients
Source: PLoS One. 2015 May 20;10(5):e0127028. doi: 10.1371/journal.pone.0127028 (PMC4439153; doi:10.1371/journal.pone.0127028)
Supplement: S3 Table — Two-group significance analysis of microarrays (SAM) between monocytes from patients with metastatic breast cancer (MBC) and sepsis [MBC / Sepsis] compared to monocytes from healthy controls [HC] (excluding tuberculosis patients and the breast cancer patient that clustered with healthy controls). The table specifies the 217 genes with significantly lower expression in [MBC / Sepsis] as compared to HC (FDR < 0.05) and relevant pathways as identified by gene ontology (DAVID). Genes of special interest in MDSCs and monocyte reprogramming are highlighted in red. (PDF) [file pone.0127028.s013.pdf]

Table S3. Two-group significance analysis of microarrays (SAM) between monocytes from patients with metastatic breast cancer (MBC) and sepsis [MBC / Sepsis] compared to monocytes from healthy controls [HC] (excluding tuberculosis patients and the breast cancer patient that clustered with healthy controls). The table specifies the 217 genes with significantly lower expression in [MBC / Sepsis] as compared to HC (FDR < 0.05) and relevant pathways as identified by gene ontology (DAVID). Genes of special interest in MDSCs and monocyte reprogramming are highlighted in red.

Gene expression lower in [MBC / Sepsis] as compared to HC			
**Cluster**	**Gene symbol**	**GO term**	**P-value**
Green	HLA-DRB6, HLA-DRB4, LOC649143, BEXL1, PON2, AKR1B1, CLEC10A, SDF2L1, NBPF11, LOC651149, HES4, VENTX, KLF4, RPL22, CNIH, POLR1C, ZDHHC1, CD86, CA2, TGIF1, ACP1, ATBF1, LOC440359, LOC642817, LOC645436, LYRM2, LOC100131609, ITGB1, FNTA, LOC648210, ASAHL, MAP4K1, LOC648024, ASAHL, PON2, CXXC5, CRTAP, NMD3, TOMM20, ATP1B3, LOC645691, PRNP, PRKRA, PPRC1, HNRPA1L-2, BXDC2, LOC728732, LOC399804, LOC645385, RAB28, LOC100131609, LOC728643, LOC402112, LOC100132528, C3orf38, LOC644877, LOC100133372	GO:0010605~negative regulation of macromolecule metabolic process<br>GO:0010629~negative regulation of gene expression<br>GO:0042127~regulation of cell proliferation	0.021<br>0.024<br>0.028
Pink	ARL5B, JUN, ARL4A, HIF1A, TNFAIP3, LOC728835, CCL4L1, CCL3, CCL3L1, CCL3L3, CXCR4, TAGAP, IL1B, NR4A2, CD83, DUSP2, IL8, SLC25A24	GO:0007626~locomotory behavior<br>GO:0006935~chemotaxis<br>GO:0006952~defense response	1.7E-7<br>3.4E-7<br>1.0E-6
Light blue	CD44, TRA2A, MLL5, KLF6, BCLAF1, C13orf15, LOC728755, TFIP11, PMAIP1, NCRNA00120, UFM1, HNRPH1, DNAJB14, FOSB, GSDML, TMEM137, LOC100134648, HNRNPH1, ETV3, LOC100133840, HBEGF, ARPC3, DUSP6	GO:0005654~nucleoplasm<br>GO:0031981~nuclear lumen<br>GO:0006397~mRNA processing	0.003<br>0.020<br>0.048
Purple	VEGFA, SF3B1, AHR, SMAD7, OLIG1, CRTAP, LOC642033, CLEC2D, C9orf21, RPLP0, VPS41, ARL4A, SON, TCEA1, ZSWIM4, LOC644063, ING3, BCAP29, ITGB1, MATR3, OSM, DDIT3, CD1C, IER3, ARRDC3, LOC729423, LOC440345, C7orf40, EBI2, LOC100131831, LOC441131, LOC729841, GFRA2, SBDSP, LOC442609, FCER1A	GO:0048534~hemopoietic or lymphoid organ development<br>GO:0002520~immune system development<br>GO:0030097~hemopoiesis	8.9E-4<br>0.001<br>0.007
Orange	ATP5F1, PRNP, C6orf48, SNURF, KLF11, CDKN1A, SON, CTNNB1, DUSP5, RIPK2, SLC25A36, MOAP1, FEM1B, KLF10, GRPEL1, MGC14376, PIM3, NXT1, IER5, SDHALP1, TIPARP, ETNK1, BZW1, CCT6A, LOC388272, ZMYM5, EAF1, YTHDC1, WDR43, LBR, ETNK1, TNF, USP36, ID2, TMEM170, LOC100132418, BCL11A, C14orf43, SBDS, BHLHB2, CASC4, LOC100132761, MCL1, C9orf72, SYNCRIP, DDX21, ILF3, CD69, PLDN, LOC653226, CSNK1A1, C3orf38, LOC644086, ZAK, SGK1, LOC644860, CYCSL1, LOC388275, MPEG1, EIF4G2, PTS, ATF3, ARL4A, CACYBP	GO:0031974~membrane-enclosed lumen<br>GO:0070013~intracellular organelle lumen<br>GO:0042981~regulation of apoptosis	1.8E-6<br>5.5E-6<br>1.3E-4
