# Supplementary material for: Systemic Monocytic-MDSCs Are Generated from Monocytes and Correlate with Disease Progression in Breast Cancer Patients
Source: PLoS One. 2015 May 20;10(5):e0127028. doi: 10.1371/journal.pone.0127028 (PMC4439153; doi:10.1371/journal.pone.0127028)
Supplement: S4 Table — Two-group significance analysis of microarrays (SAM) between monocytes from patients with metastatic breast cancer (MBC) and sepsis [MBC / Sepsis] compared to monocytes from healthy controls [HC] (excluding tuberculosis patients and the breast cancer patient that clustered with healthy controls). The table specifies the 126 genes with significantly higher expression in [MBC / sepsis] as compared to HC (FDR < 0.05) and relevant pathways as identified by gene ontology (DAVID). Genes in bold are the 37 significantly differentially expressed genes (SAM, FDR<0.05) between 1) monocytes from patients with [MBC / sepsis] and 2) monocytes from [HC / tuberculosis]. Genes of special interest in MDSCs and monocyte reprogramming are highlighted in red. (PDF) [file pone.0127028.s014.pdf]

Table S4. Two-group significance analysis of microarrays (SAM) between monocytes from patients with metastatic breast cancer (MBC) and sepsis [MBC / Sepsis] compared to monocytes from healthy controls [HC] (excluding tuberculosis patients and the breast cancer patient that clustered with healthy controls). The table specifies the 126 genes with significantly higher expression in [MBC / sepsis] as compared to HC (FDR < 0.05) and relevant pathways as identified by gene ontology (DAVID). Genes in bold are the 37 significantly differentially expressed genes (SAM, FDR<0.05) between 1) monocytes from patients with [MBC / sepsis] and 2) monocytes from [HC / tuberculosis]. Genes of special interest in MDSCs and monocyte reprogramming are highlighted in red.

**Gene expression higher in [MBC / sepsis] as compared to HC**			
**Cluster**	**Gene symbol**	**GO term**	**P-value**
Grey	HBB, HBA1, HBA2, IFIT3, ZBP1, LOC643332, ETS1, CCDC109A, C3AR1, SAMSN1, BCL2A1, **MIR1974**, MS4A4A, E2F2, LOC391769, BATF, TMEM70, **RNASE3**, TNFAIP8L2, MLSTD1, PPIG, FBXO38, FPRL1, C1orf25, RNASEL, ST8SIA4, KBTBD7, CEBPE, PPP2R2A, RRM2B, DHRS9, NRBF2, ANKRD22, **NKG7**, S100A12, NFE2, LOC100129243, ADCY3, FADD, AMY1B, ST8SIA4, MFAP1, SP3, WDR44, RAB33B, CRSP3, NLRC4, LOC728006, TLR8, GPR30	GO:0006952~defense response<br>GO:0042742~defense response to bacterium<br>GO:0009617~response to bacterium	5.9E-4<br>0.003<br>0.01
Red	**OLFM4**, **CHI3L1**, CAMP, **LOC653600**, **LOC728358**, DEFA3, DEFA1, **PGLYRP1**, **CEACAM8**, **CD24**, **LCN2**, **TCN1**, **CEACAM1**, CLC, TLR1, **TFF3**, **OLR1**, LTF, MYB, ATP8B4	disulfide bond<br>GO:0005576~extracellular region<br>GO:0006952~defense response<br>GO:0009617~response to bacterium	1.1E-7<br>1.1E-6<br>1.6E-7<br>2.2E-6
Dark blue	RETN, STOM, GPR84, NUSAP1, TSPAN2, **CTSG**, **ELA2**, **DEFA4**, **MPO**, **CEACAM6**, **BPI**, **AZU1**, DACH1, **CYP4F3**, **ANXA3**, **MS4A3**, **COL17A1**, HP, ORM1, RNF24, **CST7**, LRG1, MMP25, **MMP9**, **ARG1**, **S100P**, **IL18RAP**, **CA4**, **Rgr**, C5orf32, ALOX5AP, IFITM1, **TNFAIP6**, AQP9, ACSL1, SLC26A8, VNN1, N4BP1, HLX1, B4GALT5, TTRAP, RNF149, C7orf25, ZNF398, ALPK1, ACTN1, DYSF, ASF1B	GO:0006952~defense response<br>GO:0006954~inflammatory response<br>GO:0009611~response to wounding<br>disulfide bond<br>antibiotic	8.6E-6<br>0.003<br>0.004<br>9.6E-5<br>6.1E-4
